# The Biosynthesis Related Enzyme, Structure Diversity and Bioactivity Abundance of Indole-Diterpenes: A Review

**DOI:** 10.3390/molecules27206870

**Published:** 2022-10-13

**Authors:** Yong Hou, Meiying Chen, Zhaocui Sun, Guoxu Ma, Deli Chen, Haifeng Wu, Junshan Yang, Yihang Li, Xudong Xu

**Affiliations:** 1 Yunnan Key Laboratory of Southern Medicine Utilization, Yunnan Branch, Institute of Medicinal Plant Development, Peking Union Medical College and Chinese Academy of Medical Sciences, Jinghong 666100, China; 2 Key Laboratory of Bioactive Substances and Resource Utilization of Chinese Herbal Medicine, Institute of Medicinal Plant Development, Peking Union Medical College and Chinese Academy of Medical Sciences, Beijing 100193, China

**Keywords:** indole diterpene, synthase, bioactivity, structure–activity relationship

## Abstract

Indole diterpenes are a large class of secondary metabolites produced by fungi, possessing a cyclic diterpenoid backbone and an indole moiety. Novel structures and important biological activity have made indole diterpenes one of the focuses of synthetic chemists. Although the discovery, identification, structural diversity, biological activity and especially structure–activity relationship of indole diterpenes have been reported in some papers in recent years, they are absent of a systematic and comprehensive analysis, and there is no elucidation of enzymes related to this kind of natural product. Therefore, it is necessary to summarize the relevant reports to provide new perspectives for the following research. In this review, for the first time, the function of related synthases and the structure–activity relationship of indole diterpenes are expounded, and the recent research advances of them are emphasized.

## 1. Introduction

Indole diterpenes are a large class of secondary metabolites produced by fungi, such as aflatrems, janthitrems, lolitrems, paspalitrems, penitrems, shearinines, sulpinines and terpendoles, all possessing a cyclic diterpenoid skeleton derived from geranylgeranyl diphosphate (GGPP) and an indole moiety rooted in tryptophan [1,2]. On this basis, different functional groups or ring systems enhance the structural diversity of this kind of metabolite. More than 100 indole diterpenes have been isolated from fungi, of which one-third originated from endophytic fungi, whereas indole diterpenes from plants are rarely reported. The number of indole diterpenes has been increasing with the research in recent years. Although indole diterpenes play an important role in economic production, pest control and ecosystem protection, most of these, assuming paspaline-derived indole diterpenes, represent various hazards towards the health of humans and beasts [1]. Some the play part of a channel antagonist, causing dysfunction of the motor system, including tremors and seizures [3]. It was found that the *Festuca argentina* sheaths collected from pastures contain indole diterpenoid alkaloids, which can cause poisoning in goats [4]. These force us to warn about the impact of such natural products in our daily lives. In contrast to the previous studies on mycotoxins, today, indole diterpenes have been more studied regarding their anticancer and insecticidal activities. Certain indole diterpenes are even found as potential candidates of novel antitumor drugs, such as penitrem A [5,6]. Moreover, some related synthases of indole diterpenes belong to drug metabolizing enzymes of phase I, which offer the possibility to apply it to clinical research.

Previous studies have found that GGPP and tryptophan are major metabolic precursors of biosynthesis, and the first gene cluster cloning of indole diterpenes, the *Penicillium* gene cluster for paxilline biosynthesis, was realized by a plasmid insertion mutation and chromosome walking [7]. In recent years, genome prediction, gene mapping and enzyme cloning related to indole diterpenoid synthases have been completed gradually, and more than fifteen indole diterpenoid biosynthetic gene clusters have been identified [1,8]. At present, the preparation of indole diterpenes has been studied to optimize the process for better applications in production.

In addition, novel structures and important biological activities have made indole diterpenes receive more attention, and studies regarding derivatives of indole diterpenes have contributed to finding new drugs. In this review, almost all natural indole diterpenes reported, especially the new compounds discovered recently, were collated and listed. The compounds were reclassified according to their chemical structural correlation and sources. Through further analysis and summary, the structure–activity relationship aimed towards every class of indole diterpenes is presented comprehensively, which is instructive to design and develop more new pharmaceutical molecules. Overall, the purpose of this review is to continue to cover the previously reported literature, to expound the function of related synthases and to emphasize the research advances of structure diversity as well as the structure–activity relationship of indole diterpenes from a new point of view.

## 2. Indole-Diterpenoid-Biosynthesis-Related Enzymes

In recent years, advances in genomics, molecular biology and natural product technology have allowed researchers to break through the limitations of previous hosts. Hence, the genes and the mechanism of biosynthesis of natural products have been systematically and deeply studied in heterologous system. Up to now, there have been three key enzyme related to indole diterpenoid biosynthesis, namely prenyltransferase (PT), flavin-dependent monoxygenase (FMO) and terpene cyclase (TC).

### 2.1. Prenyltransferase (PT)

Traditionally, enzymes are considered to have strict substrate and reaction specificity. However, many enzymes have been found to catalyze substrates or reactions that are different from their “natural” ones, i.e., “heterosis” [9]. Prenyltransferases are a class of soluble proteins that catalyze different isopentenes transfer reactions on different substrates and that are involved in the biosynthetic pathway of indole diterpenes. FPP is converted to GGPP by LtmG and PaxG prenyltransferases, and it is further transformed into 3-geranylgeranylindole, an intermediate of classical indole diterpene paspaline, under the role of LtmC, TerC and PaxC prenyltransferases. The prenyltransferases can catalyze the isopentenylation of indole diterpenes either in the last step of biosynthesis or in the formation of a ring structure by binding with oxidase [10]. Prenyltransferases are derived from microorganisms and reactive heteropetropes. Predictive indole prenyltransferase genes have been found in the genomic sequences of different fungal species [11].

Indole prenyltransferase has tryptophan aminopeptidase activity, which strengthens the relationship between them. All indole prenyltransferases only accept dimethyl allyl diphosphate as an isoamyl donor [12]. When the putative gene heterologous expression is successful, the putative intermediate for NSPLD1 production is constructed using the CRISPR/Cas9 genome-editing technique [12]. The research progress of isopentenyl transferase has been summarized, including its structure and mechanism of action [13]. The introduction of isopentenyl groups in natural products not only greatly enriches the structural diversity, but it also enhances the affinity to drug targets and the bioavailability, thus improving the efficacy of drugs. As a result of the complexity and diversity of the natural products, it is difficult to carry out isopentenylation by chemical methods. Heteroplastic isopentenyl transferases provide a new strategy for this purpose.

### 2.2. Flavin-Dependent Monoxygenase (FMO)

It is a group of microsomal enzymes dependent on flavin adenine dinucleotide (FAD), reducing nicotinamide adenine dinucleotide phosphate (NADPH), and molecular oxygen, as an important liver drug and chemical foreign body metabolic enzyme, can catalyze the oxidation of nitrogen, sulfur, phosphorus, selenium and other nucleophilic heteroatom compounds. As monooxygenases, they are thought to control oxygenation by transferring a molecule of peroxanthine [14]. This study examined the function, structure and distribution of 374 flavin-dependent proteins in the genomes of 22 archaea, fungi, protozoa and eukaryotes, and it found that more than 90% of flavin-dependent enzymes are oxidoreductase [15]. FMO is the key enzyme of indole diterpenoid biosynthesis. The related enzymes of indole diterpenoid biosynthesis have been found, including LtmM [16], TerM [17], PaxM [7], etc. The above-mentioned 3-geranylgeranylindole, which is synthesized in the presence of PT, is further catalyzed by FMO to produce paspaline as the precursor of many indole diterpenes. In addition, FMO is widely found in biological processes, including drug detoxification, the biodegradation of environmental aromaticity and the biosynthesis of antibiotics and iron carriers [18]. NADPH and O_2_ are used as co-substrates for the reaction, in which an oxygen atom is inserted. FMO utilizes NADPH to reduce the general cycle of flavin. The reduced flavin reacts with O_2_ to form an intermediate of C4a-(water) peroxflavin [18].

### 2.3. Terpene Cyclase (TC)

The backbone of diterpenoid products is catalyzed by TC with GGPP (C20) as a substrate. A multitask chimeric terpene synthetase EvVS with a TC and a PT domain from the genome of the polychromatic fungus *Ericella variecolor* has been identified, and the TC domain produces the diterpene **1** and the sesterterpene **2** from GGPP and GFPP [19]. Structural information, such as TC, is essential for understanding catalytic mechanisms and guiding the study of the enzyme engineering of biocatalysts [20]. The role of cyclase in the biosynthesis of indole diterpenes is to make the uncycled part of the ring, coacting with FMO to catalyze the formation of paspaline.

### 2.4. Cytochrome P450 Monooxygenase (CYPS)

**Figure 1:**
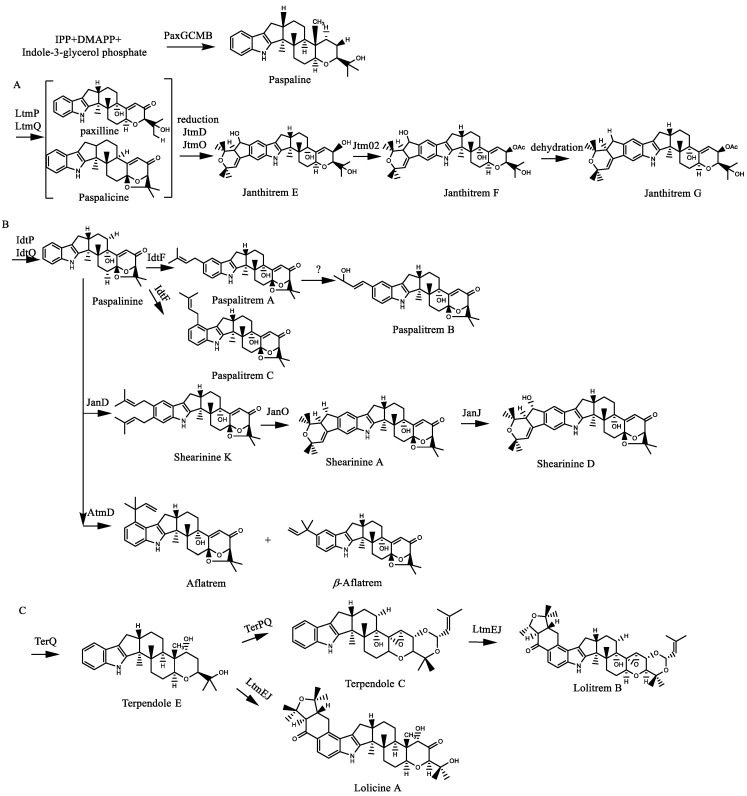
Biosynthetic pathways for classical paspaline-derived indole diterpenes (the initials stand for related enzymes). (**A**) The synthesis pathway of Janthitrem analogues in response to the corresponding Jtm enzyme. (**B**) The synthesis pathway of Paspalitrem, Shearinine and Flatrem analogues in response to the corresponding Idt, Jan and Atm enzymes. (**C**) The synthesis pathway of Terpendole, Lolicine and Lolitrem analogues in response to the corresponding Ter and Ltm enzymes.

The cytochrome P450 superfamily is a large family of enzyme proteins, widely distributed in different boundaries. In plants, CYPS is involved in the synthesis and modification of many pathways that have important functions at different stages of plant development, and it catalyzes the monooxygenation of a diverse array of xenobiotic and endogenous compounds [21]. This includes the biosynthesis and degradation of a wide range of compounds involved in various physiological responses, such as signaling and defense, organ patterning and the biosynthesis of structural polymers [20]. It belongs to monooxygenase, also known as mixed function oxidase and hydroxylase, because its reductive absorption peak is at 450 nm. P450 enzymes involved in known indole diterpenoid biosynthetic pathways include LtmQ, LtmP, TerQ, Terp, LTMK, Terk, LtmJ, etc. P450 monooxygenase can catalyze the three-step oxidation of a diene intermediate to form a 5/6 bicyclic system [22]. In addition, they can catalyze the oxidative coupling of indole to form the dimer. On this basis, it was proposed that the P450/NADPH-P450 reductase system can catalyze the oxidation of indole to produce a variety of products [23]. The biosynthetic pathway of indole diterpene is summarized in Figure 1 [17,22,24,25,26,27,28,29,30,31,32,33,34,35,36,37,38]. The diagram shows the classic paspaline-derived indole diterpenoid biosynthetic pathway, and non-paspaline-diterpenoid compounds are mostly new compounds with low structural regularity. Thus, their biosynthetic pathway remains to be studied.

## 3. Chemical and Structural Diversity of Indole Diterpenes

**Figure 2:**
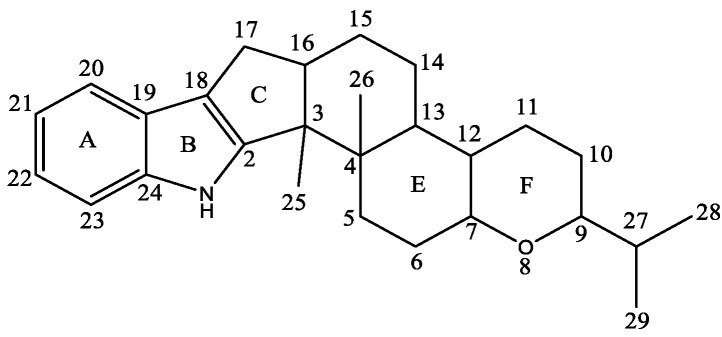
Indole diterpene skeleton.

According to statistics, over one hundred indole diterpenoid compounds have been isolated and elucidated from a variety of fungi and plants up to now. They can be categorized into paxilline- and non-paxilline-type compounds, whose skeleton structure is illustrated in Figure 2. Paxilline-type compounds feature an indole ring fused to a tetracyclic diterpene, as the core skeleton can be further classified into several subtypes, including paxilline, penitrems, lolitrems, janthitrems/shearinines, terpendoles and others [39]. In addition, non-paxilline-type compounds, as the name suggests, lack the paxilline-like core scaffold replaced by some rearranged diterpenoid rings binding with the indole moiety. They are also grouped into the following subsets: aflavinines, emindoles, eujindoles, nodulisporic acids, nominine/penicilindoles, anthcolorins, penerpenes and others.

### 3.1. Paxilline-Type Indole Diterpenes

#### 3.1.1. Paxilline

As far back as the 1970s, paxilline (**1**) was found in the cultures of the fungus *Penicillium paxilli* Bainier, initially presenting a non-linear array of six rings composed by a diterpene-derived tetracyclic scaffold of 19 carbons and an indole portion [39,40]. Afterwards, a large number of paxilline derivatives had to be obtained from various fungi in succession, such as compounds **2** and **3** from the mycelium of *Emericella striata* [41,42]; **4** from *Acremonium lolii*; paxinorol (**16**) and **13** from *Eupenicillium shearii* [25,43]; **5** and **6** from *P. paxilli* [44]; **8**, **9** and **14** from *P. camemberti* OUCMDZ-1492 [45]; **7** from *Penicillium* sp. CM-7 [46]; **15** from *Aspergillus flavus* OUCMDZ-2205 [47]; **12** and **19** from *Penicillium* sp. ZO-R1-1 [48]; and PC-M5′ (**32**) and PC-M6 (**33**) from *P. crustosum* THOM [49]. Paxilline was reduced to afford two diastereoisomeric allylic alcohols, α-paxitriol (**10**) and β-paxitriol (**11**) [50]. In 1990, the biotransformation of paxilline was investigated in sheep bile, producing a deoxygenated derivative, compound **28**, in which the 2,3 double bond of indole transformed into an eight-membered ring [51]. We also noticed that there was a novel indole diterpenoid derivative, namely secopaxilline A (**17**), possessing a unique carbon–nitrogen bond cleavage skeleton isolated from *P. camemberti* OUCMDZ-1492. Furthermore, it was generated from compound **18**, the intermediate synthesized by paxilline through an optimized one-pot process, with a 45% overall yield [52]. A series of paxilline analogs were performed by chemical modifications, including compounds **20**–**24** [53]. In 2009, penijanthine A and B (**25**, **27**), containing an equal backbone with paxilline, were isolated from *P. janthinellum* IFM 55557 and *P. crustosum* YN-HT-15, respectively [54,55]. Compound **26**, as a derivative of penijanthine A, was produced by *Penicillium* sp. CM-7 [46]. Additionally, extracts from the sclerotia of *Aspergillus sulphureus* yielded sulpinines A-C (**29**–**31**), one of which (sulpinine C), possessing an eight-membered-ring lactam, was an analog of compound **28** [56]. In 2009, epipaxilline (**34**), with a unique stereogenic at C-9, was discovered in the marine-derived fungus *Penicillium* sp. KFD28 [57].

**Figure 3:**
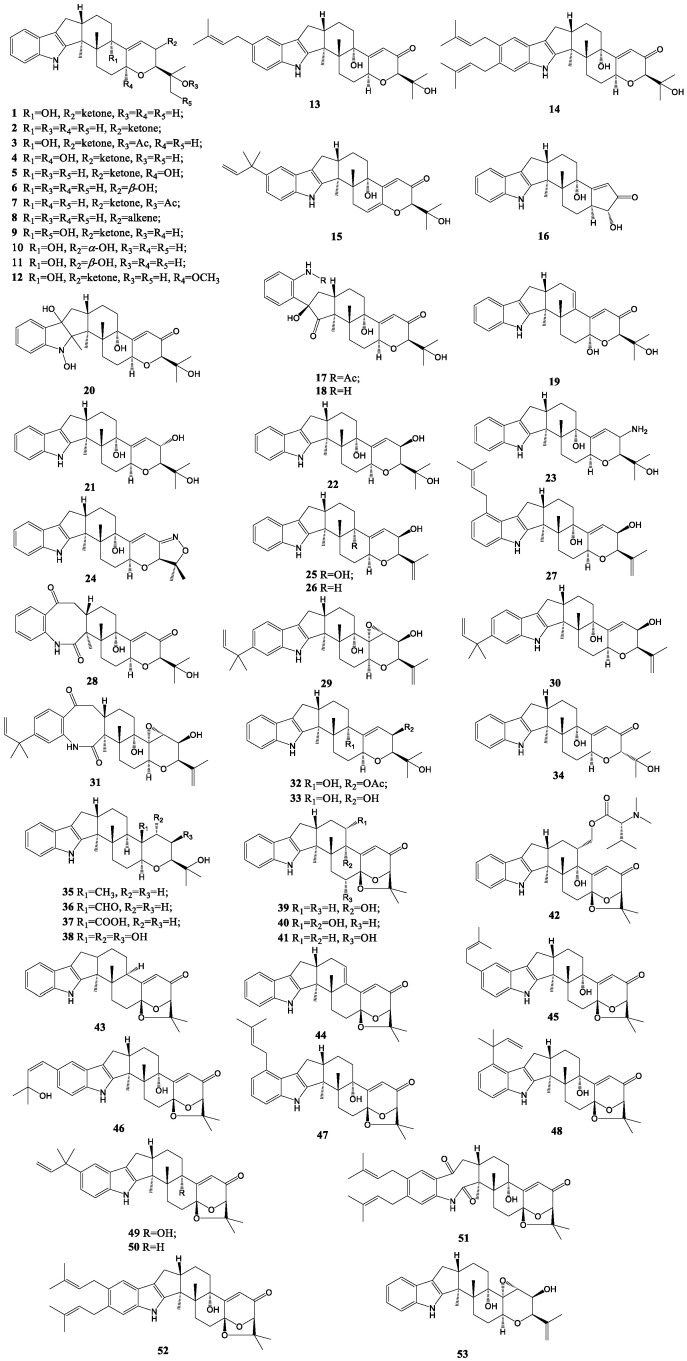
Paxilline-like indole diterpenoid compounds (**1**–**53**).

The formation of ether linkage between C-7 and C-27 in paspalinine (**39**) or paspalicine (**43**) was quite distinct from paxilline. In addition, paspalinline had a hydroxyl group at C-13 in comparison with paspalicine. Even though there was a report in 1966 that paspaline (**35**) and paspalicine are the metabolites of Clauiceps paspali, their detailed stereostructures were not established for fourteen years [58]. Paspaline B (**36**) isolated *from P. paxilli* Bainier differs from paspaline in the substitution of the aldehyde group at C-12 [59]. From *P. camemberti* OUCMDZ-1492, two paspaline analogs designated as compound **37** and **38** were obtained [45]. Compound **40**–**42** as paspalinine derivatives were separated from the sea-anemone-derived fungus *Penicillium* sp. AS-79 and *Aspergillus nomius*, respectively [60,61]. Paspalinine, along with three analogs, paspalitrems A-C (**45**–**47**), containing an additional isoprene or hydroxyisoprene unit attached to the indole ring at different positions, were also isolated and identified as metabolites from Claviceps paspali [62,63]. In 2019, a new paspalinine derivate, paspalinine-13-ene (**44**), was discovered in a chemical investigation of the endophyte *Penicillium* sp. ZO-R1-1 [64]. Aflatrem (**48**) and β-aflatrem (**49**), both produced by *Aspergillus flavus*, were paspalinine-extended by a reversed isopentenyl group attached to the aromatic ring at C-20 and C-21, respectively [65]. Additionally, 13-deoxy-β-aflatrem (**50**) was isolated from *Aspergillus flavus* OUCMDZ-2205 [47]. Herein, paspaline, paspalinine, paspalicine, paspalitrems and aflatrem, along with their derivatives, were assorted together on account of their identical backbone structures with paxilline. According to our viewpoint, shearinines J and K (**51**, **52**) isolated from an endophytic *Penicillium* sp. (strain HKI0459) should also be a part of this class rather than janthitrems, in view of their comparable backbone with paspalitrems [66]. In addition, rhizovarin F (**53**) from the fungus Mucor irregularis QEN-189 was discovered in recent years [67]. All paxilline-like indole diterpenoid compounds are summarized in Figure 3.

#### 3.1.2. Penitrems

**Figure 4:**
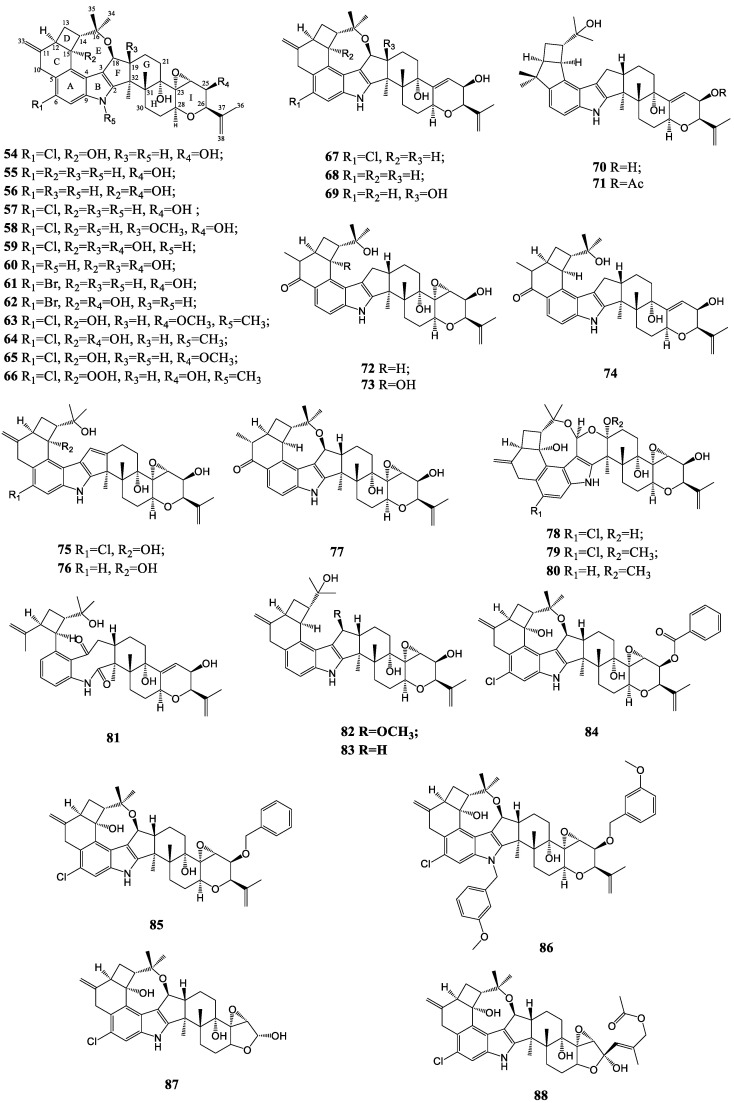
Paxilline-like indole diterpenoid compounds (**54**–**88**).

Penitrems are complex indole diterpenoid structures containing two additional isoprene units forming the cyclopentile moiety substituted into the benzylic ring and different functional groups such as chlorine, epoxide, ether bridges, etc. [39,68]. Penitrems A-H (**54**, **55**, **67**, **68**, **56**, **57**, **69**, **58**) have all been isolated from cultures of *P. crustosum*, and Amelia et al. made a greater contribution to determine their structures distinctly in the 1980s [69,70]. Secopenitrem B (**83**), from the sclerotia of *Aspergillus sulphureus*, had an intact three-ring subunit on the left-hand side compared to penitrem B, only in which the ether linkage between C-16 and C-18 was severed [56]. Compound **77**, as a penitrem analog, was also isolated from the sclerotia of *Aspergillus sulphureus* [71]. The 6-bromopenitrem B and E (**61**, **62**) were afforded by adding KBr into the fermentation broth of the fungus P. commune isolate GS20 [72]. More new penitrem analogs were semi-synthetically prepared, including compounds **63**–**66**, **84**–**86** and two lead tetraacetate (LTA) products of penitrem A (**87**, **88**) [73]. Thomitrem A and E (**75**, **76**) from extracts of *P. crustosum* Thom grown on rice had a similar framework to secopenitrem B, except for an additional 18(19)-double bond [74]. PC-M4 (**70**) obtained from *Pencillium crustosum* possessed the left-hand parts with the ring junction between the four- and five-membered rings distinguishable from penitrems. The acetylation of it afforded monoacetate (**71**), thus helping to elucidate the structure [75]. Another group of *P. metabolites*, designated as penitremones A-C (**72**–**74**), was characterized as 10-keto, 11,33-dihydro-variants of the penitrem skeleton [76]. In addition, compound **59** and its dechlorinated derivative **60** were isolated from the cultures of *Aspergillus nidulans* EN-330 [77]. Investigation of the fungus Mucor irregularis QEN-189 via genome mining resulted in the discovery of rhizovarins A-E (**78**–**82**). The presence of an acetal connected with a hemiketal or a ketal forming an unprecedented 4,6,6,8,5,6,6,6,6-fused ring system make them become the members of penitrem indole diterpenes [67]. The penitrem-like indole diterpenoid compounds are shown in Figure 4.

#### 3.1.3. Lolitrems

**Figure 5:**
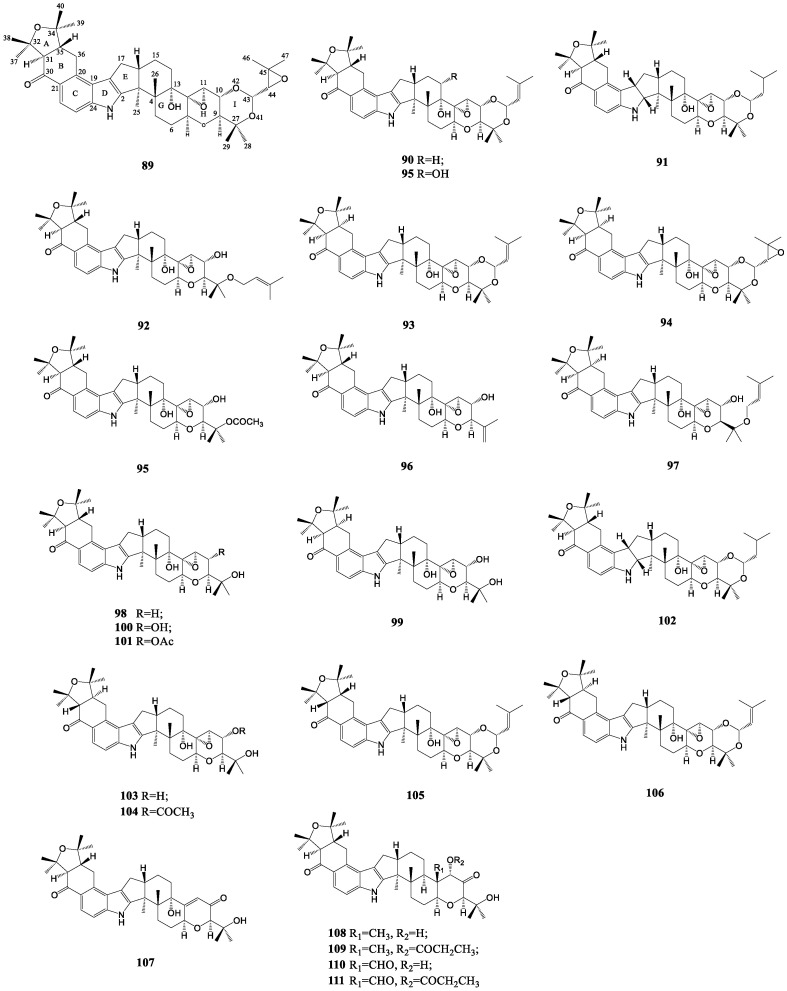
Lolitrem-like indole diterpenoid compounds (**89**–**107**).

As one of the most complex classes of indole diterpenes, the structures of them are striking because they contain more than ten chiral centers in a linear array of eight or nine contiguous fused rings. An additional mevalonate unit brings about the formation of ring I in parts of compounds [50]. Lolitrems A–C (**89**–**91**), E–H (**92**–**95**), J–N (**95**–**99**), lolilline (**107**) and lolitriols were all isolated from perennial ryegrass (*Lolium perenne* L.) infected with the endophytic fungus *Alolii* L. at the earliest time [78,79,80,81]. Therein, lolitrem A was shown to be an inseparable mixture of the 44R and 44S isomers of 44,45-epoxylolitrem C [82]. The configuration of the A/B ring junction of lolitrem B was determined with the aid of synthetic compound **102** [83]. Moreover, the acetal moiety of it was hydrolyzed to afford lolitriol (**100**), which was also the natural product of perennial ryegrass [50,84]. The base-catalyzed epimerization of lolitrem B and F afforded their 31-epimers, 31-epi-lolitrem B (**105**) and 31-epi-lolitrem F (**106**), respectively [85]. Lolilline was a naturally occurring indole diterpene belonging to the lolitrem family, with similar scaffold to lolitriol [86]. The compounds **109**, **111**, **101** and **104** confirmed that lolicines A-B (**108, 110**), lolitrem N, lolitriol and 31-epilolitrem N (**103**) are mainly natural constituents of endophyte-infected perennial ryegrass. In addition, compound **106** was identified as a minor contaminant of lolitrem B by analyzing its NMR spectra [84]. The lolitrem-like indole diterpenoid compounds are displayed in Figure 5.

#### 3.1.4. Janthitrems/Shearinines

**Figure 6:**
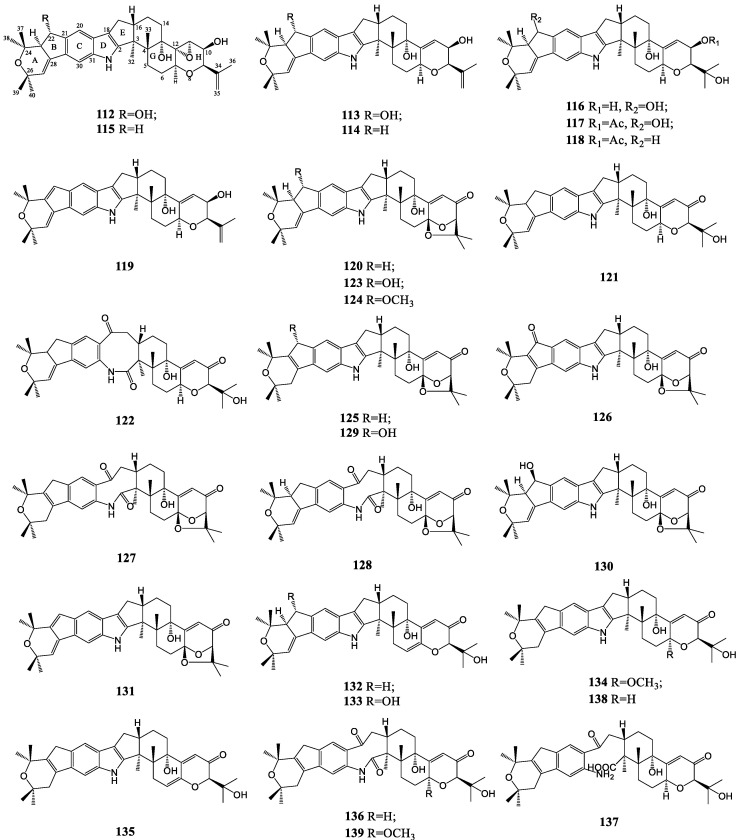
Janthitrem/shearinine-like indole diterpene compounds.

The dual rings in the linkage of indole subunit of janthitrems resemble that of pyrapaxilline (**138**), and the diterpenoid moiety is generally analogous to that of penitrems. Janthitrems A-G (**112**–**118**) were all discovered from *P. janthinellum* as fluorescent tremorgenic toxins [87,88,89]. Even though janthitrems A–D were observed firstly by high-performance liquid chromatography in the 1980s, the structures of janthitrems A and D were not determined as 11,12-epoxyjanthitrem B and 11,12-epoxyjanthitrem C exclusively, respectively, until 2018 [90]. Another derivative named JBIR-137 (**119**) was isolated from *Aspergillus* sp. FA75, featuring an additional double bond at C-22,23 distinguishable from janthitrems [91]. Pyrapaxilline from a culture of the fungus *Eupenicillium shearii* originally assumed as a paxilline analog shares an identical cyclic skeleton with janthitrems, thus belonging to the janthitrem class [92]. Shearinines, including compounds A–C (**120–122**) produced by *Eupenicillium shearii* (NRRL 3324) and D-I (**123**–**128**) isolated from endophytic *Penicillium* sp. (strain HKI0459), should also be redefined to the janthitrem class in the same light [66,93]. Among them, shearinines C, H and I all had a unique eight-membered keto-amide central ring from the cleavage of the indole C-2-C-18 bond. Of particular note, compound **130** was homonymous with shearinine E due to being reported in the same year [94]. In the screening of Candida albicans biofilm inhibitors, compound **131** with an additional double bond in ring B was isolated and characterized as a novel compound from *Penicillium* sp. isolate KS-017 [95]. In 2017, shearinines L and M (**132, 133**) produced by fungal pathogen *Escovopsis weberi* and compound **129** obtained from the fungus *Penicillium* sp. AS-79 were the newest shearinine compounds discovered to date [60,96]. Shearinines N-Q (**134**–**137**), accompanied by compound **139**, were accessed from *Penicillium* sp. ZO-R1-1, and shearinine Q was viewed as the most unique structure molecule, owing to the cleavage of the keto-amide ring [48]. These kinds of indole diterpenoid compounds are summarized in Figure 6.

#### 3.1.5. Terpendoles

**Figure 7:**
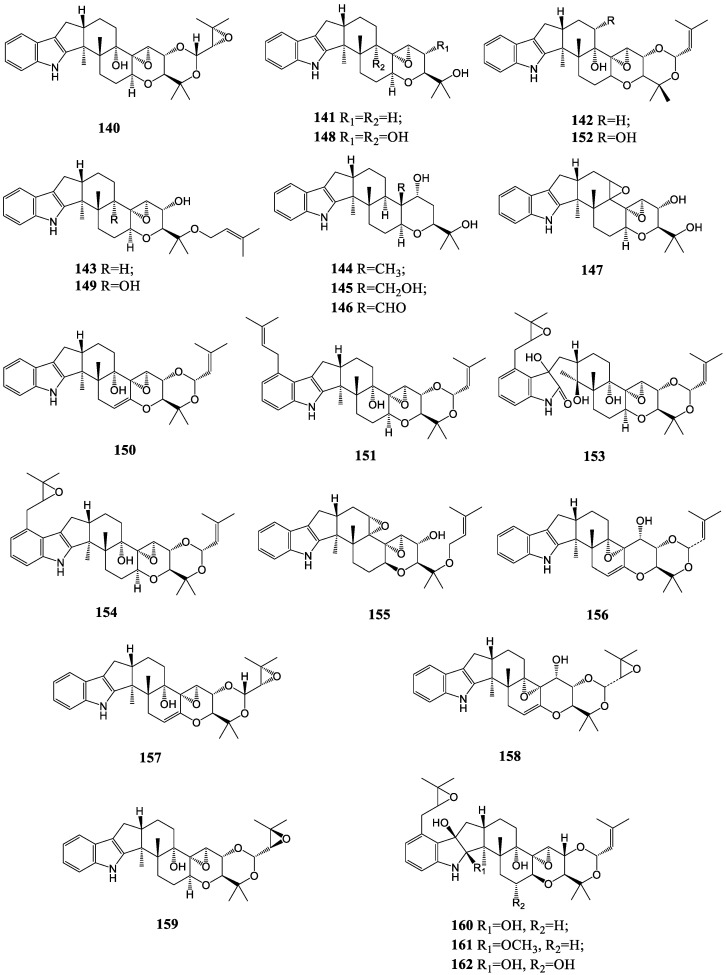
Terpendole-like indole diterpenoid compounds.

Most terpendole compounds, as shown in Figure 7, feature an additional isoprene unit jointed at the C-27 hydroxyl group, where some may further form a new ring structure on the right-hand side of molecules such as terpendoles A, C, K and L. In the 1990s, terpendoles A-L (**140**–**151**) were isolated and characterized from a new fungal strain, *Albophomayamana shiensis* FO-2546, continuously [97,98,99], whereas the terpendole M (**152**) was discovered from perennial ryegrass (*Lolium perenne*) infected with the fungus *Neotyphodium lolii* [100]. In the 2010s, compounds **156**–**158** were all found and isolated from Ipomoea muelleri and I. asarifolia according to HPLC–MS/MS analysis [101]. 2′-epi-terpendole A (**159**) was obtained from *Drechmeria* sp [102]. In the last year, new terpendole congeners N-P (**153–155**) were isolated from the fungus Volutella citrinella BF-0440, and only terpendole N contained a distinctive indolinone plus a system of four consecutive rings [103]. Moreover, new compounds designated as voluhemins A and B (**160, 161**), along with NK12838 (**162**) reported in an earlier patent, were isolated from the fungal strain *Volutella citrinella* BF-0440, which all had a characteristic isopentenyl unit attached by an epoxy group linked to an indoline moiety [104].

#### 3.1.6. Others

**Figure 8:**
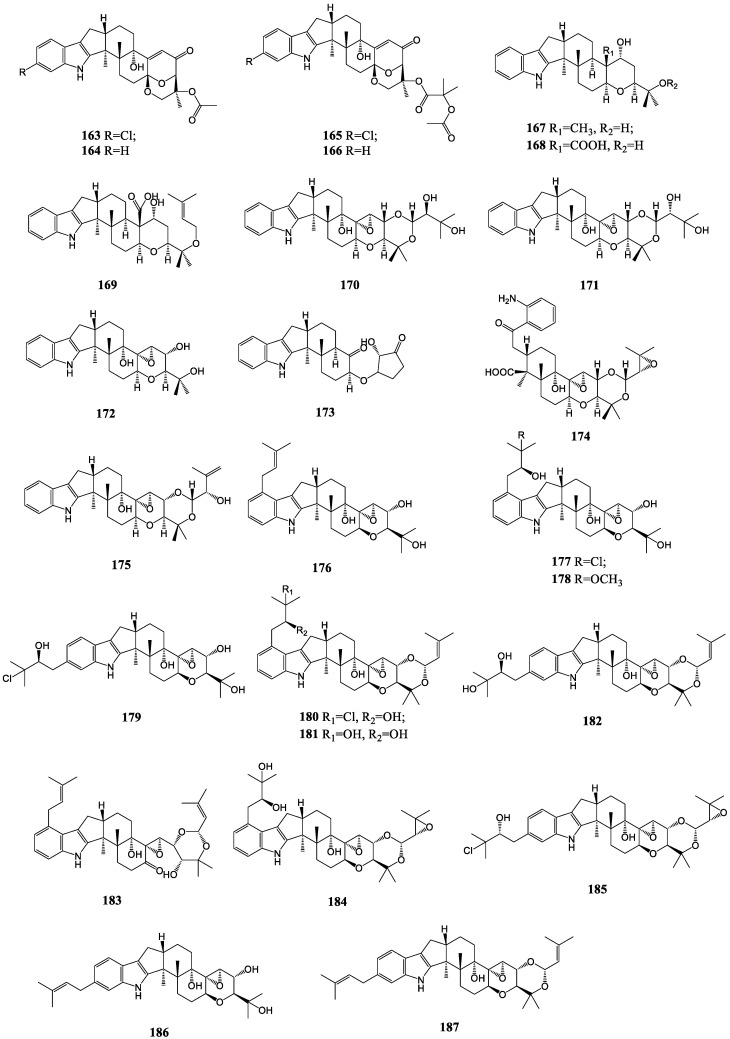
Other paxilline-type indole diterpenes.

Except for the above several classes of indole diterpenes, traditionally, there have been other novel compounds underlying the paxilline-type framework discovered in recent years (Figure 8). Asperindoles A-D (**163**–**166**) from the ascidian-derived fungus *Aspergillus* sp. KMM 4676 have unusual 1,3-dioxane rings with acetoxy groups or acetylated residues of 2-hydroxyisobutyric acid at C-27 [105]. In the fungus *Drechmeria* sp. derived from the root of Panax notoginseng, nine new indole diterpenes termed drechmerins A-I (**167**–**175**) were isolated, and parts of them (D, E, I) had an identical skeleton with terpendoles apart from drechmerin G, containing a five-membered lactonic ring linked at C-7 through an oxygen bridge, and drechmerin H, featuring a reduced indole moiety [102,106,107]. Twelve prenylated indole diterpenoid compounds, tolypocladins A-L (**176–187**), including four chlorinated metabolites, were found in the mine-soil-derived fungus, *Tolypocladium* sp. XL115. Only tolypocladin H had an unusual ketone carbonyl at position C-7, which may form the cleavage of the ether bond (C-7−O−C-9) in tolypocladin E and an oxidation reaction subsequently [108,109].

### 3.2. Non-Paxilline-Type Indole Diterpenes

#### 3.2.1. Aflavinines

**Figure 9:**
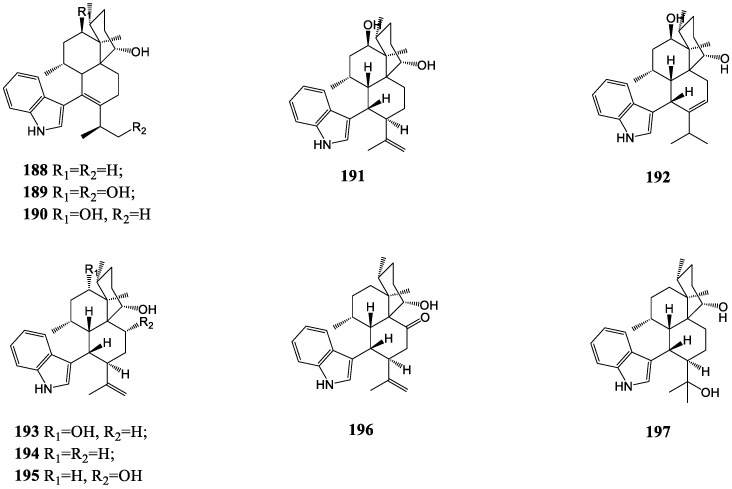
Alfavinine and its derivatives.

The discovery of aflavinine indole diterpenes has been concentrated mainly in the last century. Afavinine (**188**), produced by the fungus *Aspergillus flavus* in 1980, has an unusual framework featuring the conjugation of the extra-annular C=C with the indole moiety at position C-3 [110]. From the same fungus, other relative structural aflavinine derivatives were isolated and identified, such as compounds **189**–**192** [111,112]. Moreover, three metabolites, compounds **193**, **194** and **196**, characterized with an aflavinine ring system, were afforded by the sclerotia of the fungus *Aspergillus tubingensis* (NRRL4700) [113]. It was reported in 1995 firstly that compounds **194** and **197** were the aflavinine derivatives from a fungus *Eupenicillium crustaceum* rather than *Aspergillus* [114]. In 2019, it was first reported that cladosporine A (**195**), as the congener of aflavinine, was discovered in a fungal strain of *Cladosporium* sp. JNU17DTH12-9-01 [115]. The structures of alfavinine and its derivatives are shown in Figure 9.

#### 3.2.2. Emindoles

**Figure 10:**
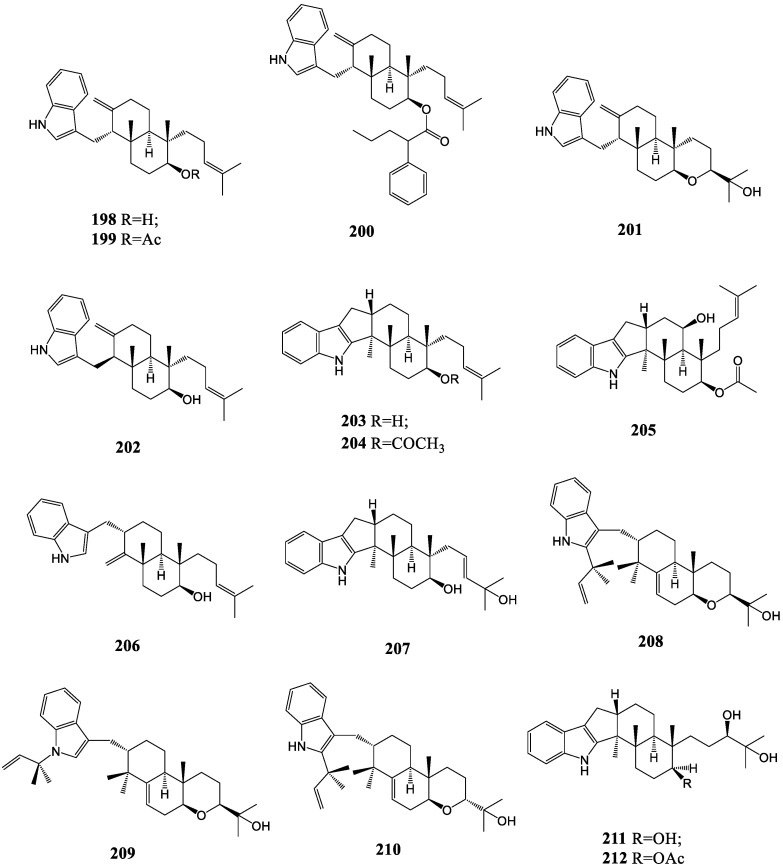
Emindole-like indole diterpenoid compounds.

In successive searches regarding the fungus *Einericella desertorurn*, emindole DA (**198**) and DB (**201**), containing an acharacteristic indol-3-ylmethyl entity with a bicyclic skeleton, were isolated and confirmed on the basis of the X-ray crystal structure determination of emindole DA’s monoacetate (**199**) and its 17-*O*-2-phenylbutyrate (**200**) [116,117]. The emindole SA (**202**) obtained from the acetone extract of *Emericella striata* was identified as a diastereoisomer of emindole DA [118]. However, the emindole SB (**203**) from the same fungus had a paxilline skeleton featuring a distinctive 4-methylpent-3-enyl group [41]. One of the emindole SB derivatives, compound **204**, was found in the investigation of the sea-anemone-derived fungus *Penicillium* sp. AS-79 [60]. In addition, the emindole SC (**205**) separated from *Aspergillus sclerotiicarbonarius* had additional hydroxyl and acetyl groups compared to emindole SB [119]. The only difference between another analog, emeniveol, (**206**) from the fungus *Emericella nivea*, and emindole DA is the conjunctive position of the bicyclic skeleton with an indole moiety [120]. Asporyzin C (**207**), as the analog of emindole SB, was separated from an endophytic fungus *Aspergillus oryzae* [121]. Emindoles PA-PC (**208–210**), bearing a 1,1-dimethyl-2-propenyl residue at C-2 or N-1 in the indole moiety, were isolated from the mycelium of *Emericella purpurea* [122]. Additionally, penijanthines C and D (**211, 212**) from *P. janthinellum* could actually be considered analogs of emindole SB because the structural difference between them was only that the 26,27-trisubstituted double bond in emindole SB was replaced by a vic-diol moiety [64]. The structures of emindole-like indole diterpenoid compounds are shown in Figure 10.

#### 3.2.3. Eujindoles

**Figure 11:**
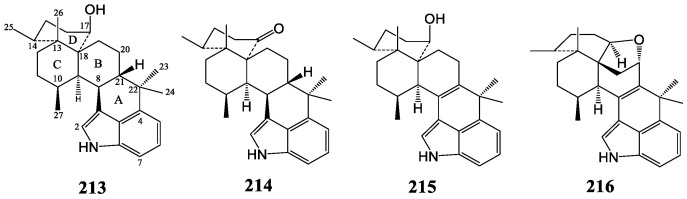
Structures of euijindole indole diterpenes.

Research on the extract of *Eupenicillium javanicum* IFM 59075 led to the isolation of compounds **213**–**216** with a hexacyclic-containing indole ring, which is illustrated in Figure 11 [123,124]. Among them, only compound **216** had a special stereochemistry structure with an additional ether ring, whose C and D rings were both in a boat conformation, and the B-C and C-D ring junctions were both cis [124].

#### 3.2.4. Nodulisporic Acids

**Figure 12:**
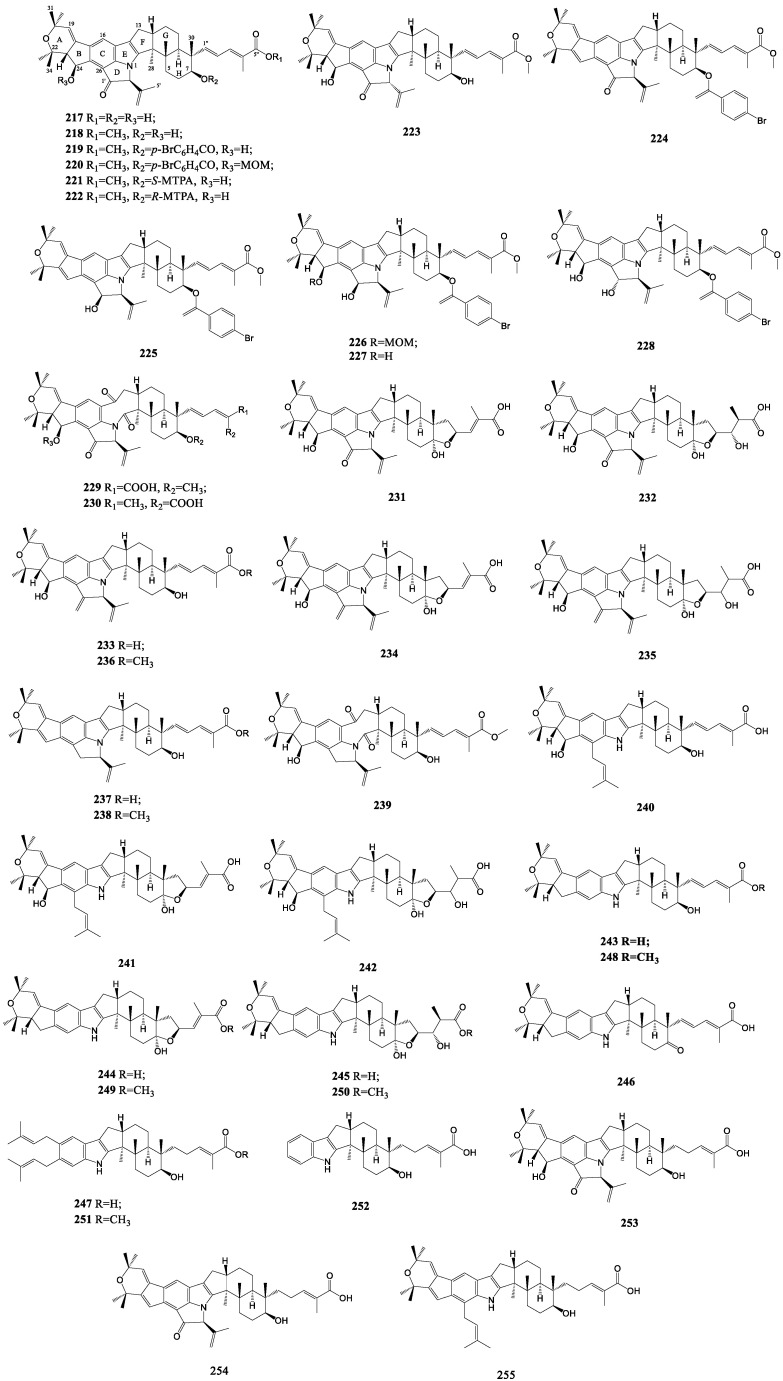
Structures of nodulisporic acid indole diterpenes.

Nodulisporic acids (Figure 12) are regarded as a new class of indole diterpenes represented by nodulisporic acid A (**217**), with an unprecedented highly strained five-membered *β*-ketodihydropyrrole ring derived from the isoprenylation of the indole moiety and a reversed ring fusion of the dihydropyran, which is purified from fermentations of *Nodulisporium* sp. (MF 5954, ATCC 74245). To elucidate its complete structure, a number of derivatives were synthesized, such as compounds **218**–**228**. Of particular note, nodulisporic acid A degraded into two oxidation products (**229**, **230**) to differ only in the dienoic acid side chain during the course of NMR experiments in CD_3_CN [125]. Successively, nodulisporic acids A1 and A2 (**231**, **232**) containing an additional five-membered tetrahydrofuran ring and a series of 1′-deoxy congeners and nodulisporic acids B, B1 and B2 (**233**–**235**) were generated from the same fungus [126,127]. Moreover, a new isolation procedure was used to obtain a decomposition product of nodulisporic acid A (**237**), which was identified by its methyl ester (**238**) given by its reaction with diazomethane. The methyl ester and the oxidized product of nodulisporic acid B (**236**, **239**) were also yielded by reacting with trimethylsilyl-diazomethane followed by normal separation [126]. The continued investigation of the fungus led to the isolation of several D-ring-opened nodulisporic acids C, C1 and C2 (**240**–**242**) [128]. Subsequently, nodulisporic acids D, D1, D2, D3 and E (**243**–**247**) from ATCC74473; nodulisporic acid F (**252**) from MF6518; and A4, Δ23-A4 and Δ23-C4 (**253**–**255**) from two other mutant cultures were accessed in research of mutant *Nodulisporium* strains. Likewise, the corresponding methyl esters of nodulisporic acids D, D1, D2 and E (**248**–**251**) were also afforded by derivative reactions. The compounds of the D series and E are all devoid of an isoprene residue at C-26 in comparison with the A series. Nodulisporic acid E has only two isoprene residues not forming the A/B rings. Nodulisporic acid F (**252**) is the simplest of all nodulisporic acids and lacks all three isoprene residues of the indole unit [129].

#### 3.2.5. Nominine/Penicilindoles

**Figure 13:**
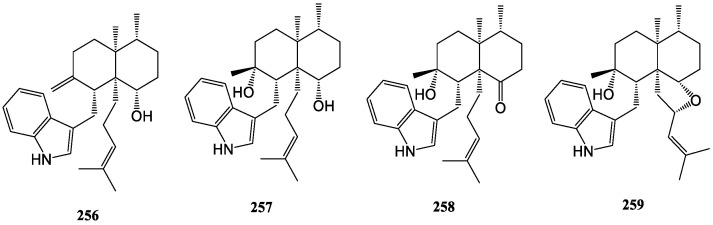
Structures of nominine/penicilindoles indole diterpenes.

Nominine (**256**) was discovered as a major organic soluble component of the sclerotia of the fungus *Aspergillus nomius* (NRRL 13137), which possesses one less ring and one more double bond compared to aflavinines [130]. In 2018, three new nominine analogs, penicilindoles A−C (**257**–**259**), were isolated from the mangrove-derived fungus *Eupenicillium* sp. HJ002. Only penicilindole C formed a distinctive five-membered ring due to the methylene at the C-22 position of penicilindole A being replaced by an oxygenated methane [131]. Their structures are displayed in Figure 13.

#### 3.2.6. Anthcolorins

**Figure 14:**
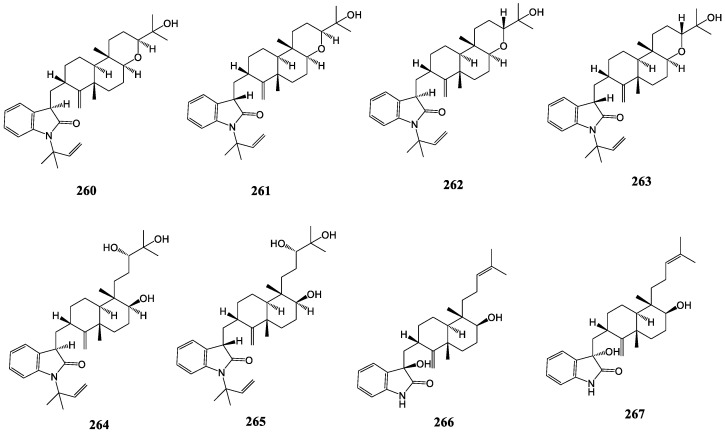
Structures of anthcolorin indole diterpenes.

Anthcolorins A-H (**260**–**267**), as shown in Figure 14, are unique tetrahydropyrane-diterpene-type metabolites with oxoindoline at C-3, originating from the endophytic fungus *Aspergillus versicolor* [132,133].

#### 3.2.7. Penerpenes

**Figure 15:**
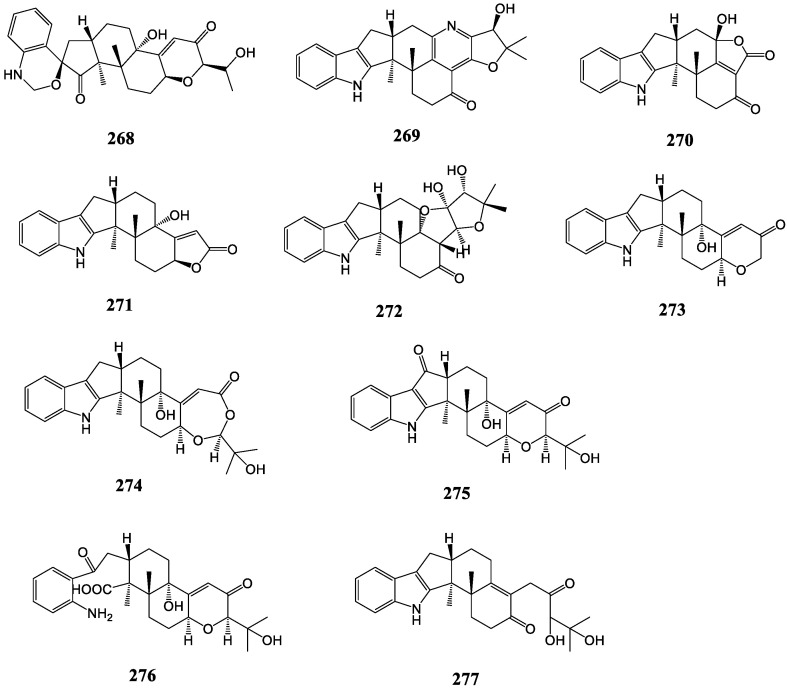
Structures of penerpene indole diterpenes.

Penerpenes (Figure 15) isolated from the marine-derived fungus *Penicillium* sp. KFD28 are representative of new skeleton indole diterpenes, of which almost all are rarely encountered in natural products. Penerpene A (**268**) is a distinctive spiro indole diterpene featuring a 1,4-dihydro-2H-benzo[d][1,3]oxazine motif. Penerpene B (**269**) has a unique pyridine-containing heptacyclicring system in indole diterpenes. Penerpenes C and D (**270, 271**) may be derived from paxilline via losing five (C-21/22/23/24/25) and four (C-22/23/24/25) carbons, respectively [134]. Penerpene E (**272**) contains a unique 6/5/5/6/6/5/5 heptacyclic ring system, and penerpene F (**273**) may be derived from paxilline by the loss of three carbons (C-23/24/25). Penerpene G (**274**) features an additional oxygen atom to form an unusual 6/5/5/6/6/7 hexacyclic ring system compared to paxilline. The structure of penerpene H (**275**) closely resembles that of paxilline, with the main difference being CH_2_-7 in paxilline replaced by a ketone carbonyl. Penerpene I (**276**) is the cleavage formation of the keto-amide ring in 2,18-dioxo-2,18-secopaxilline, essentially [135]. In addition, penerpene J (**277**) features an open terminal ring linked to an unusual △12(13) double bond position [57].

#### 3.2.8. Others

**Figure 16:**
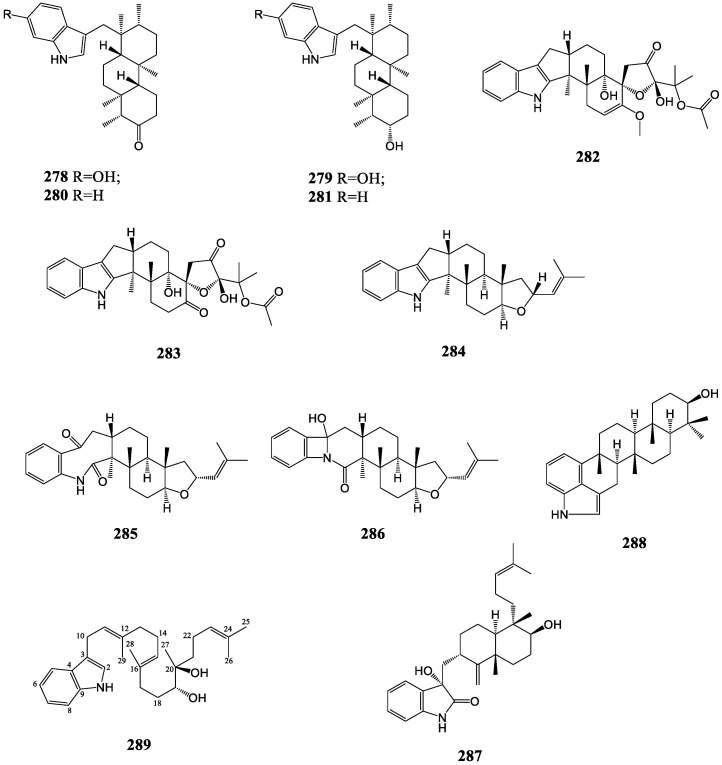
Other non-paxilline-type indole diterpenes.

Some other indole diterpenes, as shown in Figure 16, which feature diverse scaffolds have been discovered constantly. A set of new indole diterpenes named radarins A-D (**278**–**281**) were separated from the fungus *Aspergillus sulphureus*, which have a linkage between the indole moiety and the tricyclic diterpenoid structure via methylene [136]. Thiersinines A and B (**282**, **283**) were obtained from the fungus *P. thiersii* NRRL28147, containing an unprecedented spirocyclic subunit, which consisted of a rare enol methyl ether unit lacking a conjugated carbonyl unit [137]. A particular indole diterpenoid compound, JBIR-03 (**284**), originating from the fungus *Dichotomomycescejpii var. cejpii* NBRC103559, possesses a terminal ring system that consists of a rare five-membered furan ring [138]. A dioxoindole derivative of JBIR-03, asporyzin A (**285**), accompanied with its rearranged derivative, asporyzin B (**286**), was isolated from the endophytic fungus *Aspergillus oryzae* [121]. Penicindopene A (**287**), representing the first example of indole diterpenes featuring a 3-hydroxyl-2-indolone moiety, was obtained from the deep-sea fungus *Penicillium* sp. YPCMAC1 [139]. In a survey on the ascostromata of *Petromyces muricatus*, petromindole (**288**) was isolated and determined as a unique 3,4-disubstituted indole skeleton structure [140]. An extraordinary structure molecule without rings in its diterpenoid part was determined as compound **289**, which originated from *P. camemberti* OUCMDZ-1492 [45].

## 4. Pharmacological Properties of Indole Diterpenes and Their Structure–Activity Relationship

Indole diterpenes are a pronounced class of compounds as well-known tremorgens which lead to the disorder of “ryegrass staggers” in livestock. Moreover, most of them have been reported to possess excellent anti-cancer activity, anti-insect activity, the modulation of a high-conductance Ca^+^-activated K^+^ (maxi-K, BK) channel, the inhibition of acyl-CoA with cholesterol acyltransferase (ACAT), antimicrobial activity and some other activities. Discussion on the structure–activity relationship is pivotal for the development of new clinical medicines in the future.

### 4.1. Anti-Cancer Activity

**Figure 17:**
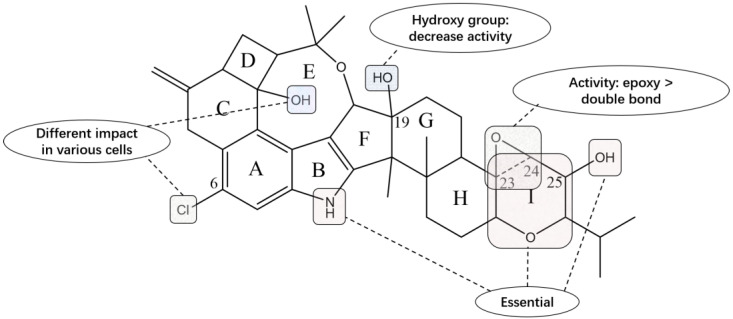
Structure–activity (anti-cancer) relationship of penitrem indole diterpenes.

Paspaline, emnidole SB and penitrems A, B, D, E and F, as well as compounds **61** and **62**, can be regarded as good inhibitors of the proliferation, migration and invasion of human breast cancer cells. Particularly, penitrem B displays the highest antiproliferative activity against MCF-7 cells. This activity results in a small decrease when the 23,24-epoxide is substituted by a double bond in penitrem B. A chlorine atom at C-6 in penitrem F, or a hydroxyl group at C-15 in penitrem E, also reduces the activity, but there is no additive effect, as expected when they both exist in penitrem A. On the other hand, penitrem A shows the highest antiproliferative activity against MDA-MB-231 cells, and the replacement of 23,24-epoxide with a double bond in penitrem D markedly leads to a reduction in activity. The lack of either chloro at C-6 in penitrem E or hydroxyl at C-15 in penitrem F decreases the activity. No additive effect has been found in penitrem B with the absence of both substitutions, which is the same as in MCF-7 cells. The introduction of a bromine atom at C-6 in penitrem B and E has small effects on the antiproliferative activity against both cell lines. Moreover, paspaline exhibits better antiproliferative activity compared to emindole SB. In a cell invasion assay, penitrem A is the indole diterpene with the highest activity among these compounds. From the comparison, we know that both chloro at C-6 and hydroxyl at C-15 are important, and the 23,24-epoxide replaced by a double bond is irrelevant to the invasive activity in the penitrem class of indole diterpenes. Compound **61** shows a substantially higher anti-invasive effect than that of its chlorinated counterpart, whereas **62** does not. Both paspaline and emindole SB barely have significant anti-invasive effects. In a screening program using 60 human tumor cell lines by the National Cancer Institute, penitrems A, B and E were evaluated for their anti-cancer activity, and compound B possessed selective activity against all cancer cells corresponding to leukemia [72]. Further study implies that these indole diterpenoid compounds inhibit cellular proliferation on MDA-MB-231 cells, which is possible via suppressing the Wnt/β-catenin pathway. Compound **63**–**66** and **84**–**88** are used to explore the structure–activity relationship of this class of indole diterpenes. Their activities are all evaluated using the above methods, and it is apparent that the pyran ring, C-25 hydroxyl and NH groups are crucial for the overall in vitro activity of penitrems [73]. The relevant structure–activity relation is presented in Figure 17.

Exploration of *M. irregularis* QEN-189 has led to 20 indole diterpenoid compounds, whose antitumor activities were all estimated against the HL-60 and A549 cell lines (Table 1) [67]. Most of the compounds showed weak or no activity towards these two cell lines aside from rhizovarins A-B, penitrems A-C, F and compound **6**. It was noticeable that the chlorinated derivatives had stronger activity than their chlorine-free counterparts. This observation hints that chlorine substitution can increase the activity against these cell lines. On the other hand, compound **6**, lacking a 13-hydroxy group and 10-keto, showed apparent activity in this assay compared to paxilline. Compound **8** was inactive, indicating the importance of 10β-hydroxy of paxilline-type indole diterpenes taking effect.

Compound **15**, **49**, **50** and paspalinine were corroborated to possess weak cytotoxicity against MCF-7 and A549 cell lines, with an IC_50_ value range of 18–30 μM. Further studies have illustrated that compound **15** and **50** are both able to arrest the A549 cell cycle in the S phase at 10 μM, and the former compound also shows PKC-beta inhibitory activity [47].

In a screening study of indole diterpenes from the endophyte *Penicillium* sp. ZO-R1-1, 16 compounds were evaluated for their cytotoxic activity toward the L5178Y, A2780, J82 and HEK-293 cell lines (Table 2) [48]. Against the L5178Y cell line, compound **5**, **44**, **19** and shearinines O-P exhibited strong cytotoxicity. Moreover, emindole SB, compound **13** and pyrapaxilline displayed mild cytotoxicity, whereas other compounds all had no activity. Toward the A2780 cell line, shearinine O, emindole SB and paspaline showed pronounced activity. The remaining compounds possessed moderate cytotoxicity, except for inactive compound **5**, **13** and **19**. From the results observed, the presence of a double bond at Δ13(14) can increase the cytotoxicity of paspalinine derivatives. However, it hardly affects the activity of paxilline derivatives. With respect to janthitrem derivatives, the double bond at Δ6(7) promotes the cytotoxicity slightly. Moreover, the substitution of a methoxy group at C-7 tends to attenuate the activity and cleavage of the keto-amide ring in the eight-membered ring skeleton of shearinine P, resulting in the loss of activity. Furthermore, most of these compounds are not sensitive towards the J82 and HEK-293 cell lines [48].

In the test for cytotoxic properties against mouse epidermal JB6 P^+^ Cl 41 cells, shearinines A, D, K and E did not show any cytotoxicity. However, shearinine E was able to inhibit the EGF-induced malignant transformation of JB6 P^+^ Cl 41 cells with INCC50 (inhibition of the number of colonies of 50) of 13 µM in the anchorage-independent transformation assay. Furthermore, shearinines A, D and E could cause apoptosis in human leukemia HL-60 cells at 100 µM with apoptosis rates of 10%, 39% and 34%, respectively, indicating that the loss of the hydroxyl group in shearinine A can decrease activity [94]. The cytotoxic activities of JBIR-137 and janthitrem B were examined against the SKOV-3 cell line, which pointed out the inevitability of the double bond at Δ22(23) for the improvement of activity in janthitrems [91].

There are also many reports about antitumor activity regarding some other types of indole diterpenoid compounds. In an investigation against human prostate cancer cell lines, asperindole A displayed evident cytotoxicity in human PC-3, LNCaP and 22Rv1 cell lines, and asperindole C showed noncytotoxicity against all three cell lines. Further studies demonstrated that asperindole A was capable of inducing apoptosis in 22Rv1 cells at low micromolar concentrations [105]. The additional acetyl group in asperindole C led to the loss of cytotoxicity. During the screening of M-phase-specific inhibitors for potential anti-cancer agents, terpendole E was found as a novel kinesin Eg5 inhibitor. In contrast, the congener terpendoles C, H and I were all inactive [141]. Radarins A and B were demonstrated to possess similar cytotoxicity against A549, MCF-7 and HT-29 cells, with ED_50_ values ranging from 0.7 to 5.5 μg/mL [136]. The cytotoxicity of penicindopene A featuring a 3-hydroxyl-2-indolone moiety was evaluated against the A549 and HeLa cell lines, which showed moderate activity [139]. Penicilindoles A and B were evaluated to exhibit cytotoxicity against the A549, HeLa and HepG2 cell lines. The activity of penicilindole A was noticeably higher than that of B, which can possibly be ascribed to the hydroxyl group rather than carbonyl moiety at the C-19 position [131]. In the cytotoxic evaluation in vitro of tolypocladins A-J against the cell lines, only tolypocladin A displayed weak cytotoxicity against all cancer cell lines and inhibited the growth and viability of the HCC cells T1224 in the patient-derived organoids model, which was attributed to the extraordinary prenylated indole nucleus [109]. JBIR-03 did not show any cytotoxic activity against the HT-1080 cell line at a concentration of 100 μM [138]. While screening the cancer cell growth inhibitory properties of anthcolorins A-F, compounds B-D were found to exhibit more potent cytotoxic activity than others, notably suggesting that the presence of the C ring existing in a boat conformation is required for strong activity (Table 3) [132]. In addition, for the oxoindolo diterepenoid epimers, the cytotoxic activity towards the HeLa cell line of 3α-epimer (anthcolorin H) was evidently higher than that of 3β-epimer (anthcolorin G), further elaborating that their cytotoxic activity in anthcolorins depends on their stereochemistry [133].

### 4.2. Tremorgenic Activity

**Figure 18:**
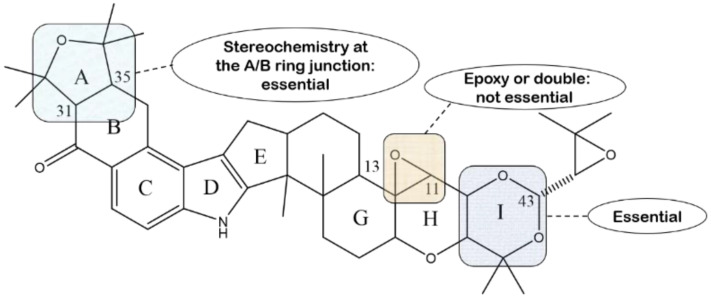
Structure–tremorgenic activity relationship of lolitrem indole diterpenes.

From the last century, a substantial amount of research has indicated that most indole diterpenoid alkaloids discovered early, particularly the lolitrems, possess anti-mammalian bioactivity for inducing the “ryegrass staggers” disorder, which is a nervous disorder in animals grazing on endophyte-infected perennial ryegrass, notably causing tremors [142]. An extensive study with respect to lolitrem A has shown that the tremorgenic activity of lolitrem A is as high and prolonged as that of lolitrem B, implying that, regardless of using an epoxy or double bond derived from the isopentenyl unit of the I ring, it has no impact on the intense tremors induced by lolitrems [82]. Lolitriol, which is synthesized using lolitrem B as a parent, is much less tremorgenic than its parent compound only due to lacking an isopentenyl unit to form the I ring [50]. Similarly, lolitrem B given to mice intraperitoneally can elicit an easily detected tremorgenic response, whereas no tremors can be detected with lolitrem E under the same conditions [80]. The moiety of the acetal-linked isoprene unit, which is only a different point between them, should be the key to triggering tremorgenic activity. In a standard mouse bioassay, lolitrem F and compound **106** were both found to have potencies and durations of action similar to those of lolitrem B, and compound **105** only caused detectable tremors [85]. The cis-fused isomers (lolitrem F and compound **105**) with an A ring protruding from the plane of the molecule differs from the tran-fused isomers (lolitrem B and compound **106**) with a relative planar in rings A-H. It is obvious that the stereochemistry of the A/B ring junction has no influence on the tremorgenic activity of the lolitrems, and only the A ring oriented on the α-face can make activity disappear [85]. The structure–activity relationship is illustrated briefly in Figure 18. It is likely that lolitrem B induces tremors in animals through the inhibition of large conductance calcium-activated potassium (BK) ion channels [90].

**Figure 19:**
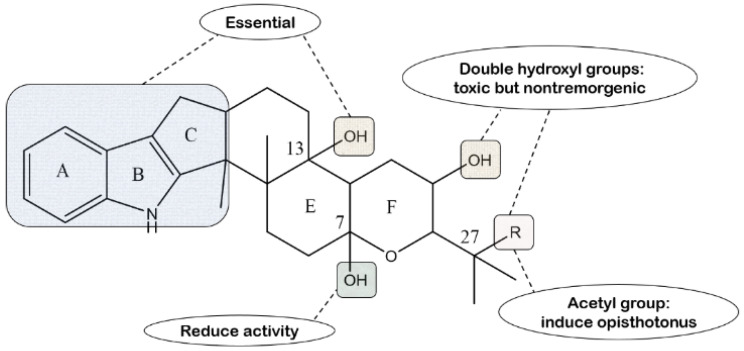
Structure–tremorgenic activity relationship of paxilline indole diterpenes.

Paxilline has been reported to induce early severe tremors in mice sustained for several hours [40,58]. Deoxygenated paxilline is biologically inactive when given to mice at ten times the dose of that used for paxilline, which suggests that the indole nucleus is essential for tremorgenic activity [51]. In addition, in the subsequent research, it has been demonstrated that paxilline is much more active than lolilline, which belongs to lolitrems [86]. Additionally, compounds **10** and **11**, both synthesized from paxilline, have no tremorgenic activity compared with their parent compounds, but they cause rough coats and lethargy in animals. Compound **11** and lolitriol can even lead to mice death in the test dose. It seems that indole diterpenes, bearing C-92-propanol and C-10 hydroxyl moieties, may be toxic but nontremorgenic [50]. Moreover, the tremorgenicity caused by compound 3 is not only almost as strong as that of paxilline, but it is also characteristically opisthotonus in mice, which is greatly relevant to the addition of an acetyl group [42]. In comparison with prominent tremorgenicity of paxilline, compound 4 does not lead to any tremors in mice under the same conditions, suggesting that the oxidative transformation of paxilline at the C-7 position significantly reduces tremorgenic activity [44]. Cole et al. reported that paspalicine and paspaline are not tremorgenicic in mice at 250 and 500 mg/kg, respectively [58]. However, paspalinine at 80 and 160 mg/kg showed noticeable clinical symptoms, similar to those for the tremorgen paxilline due to the addition of 13R-OH. It is worth noting that the presence of the tertiary hydroxy group at C-13 is required for tremorgenic activity in the indole diterpenes [62]. The structure–activity relationship is shown in Figure 19.

Based on the above, a possible metabolic pathway was proposed: a reduction in ketones in paxilline takes place in ruminants and mammals, resulting in compounds **10/11**. Lolitrem B is catalyzed via acetal hydrolysis by an enzyme or acid, so it converts into lolitriol. Thus, the two tremorgens lolitrem B and paxilline are closely related with A. Lolii-infected ryegrass may be transformed into being toxic, but nontremorgenic compounds are partially transformed by livestock [50].

**Figure 20:**
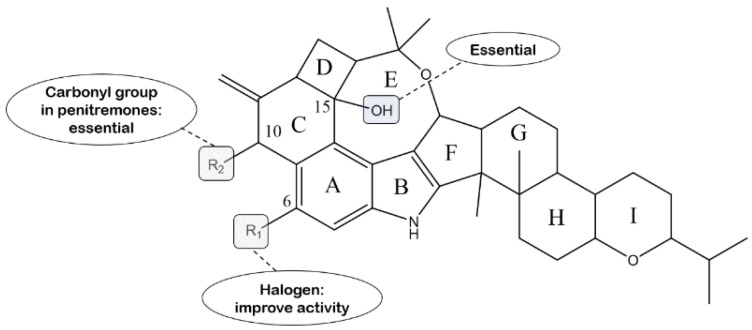
Structure–tremorgenic activity relationship of penitrem indole diterpenes.

In terms of penitrems, penitrem A is usually considered to be the most potent tremorgen of this group of indole diterpenes, which is approximately active three times more than penitrem E, implying that the chlorine atom replaced in the C-6 position can improve its activity [143]. It was reported in the year of 2013 that the calculated tremorgenic EC_50_ for penitrem A in mice is 0.6 mg/kg. Compound **62** displays comparable tremorgenic potential with its chlorinated counterpart (penitrem A), suggesting that the tremorgenicity of penitrems is hardly influenced by the type of halogen. In addition, compound **61** is a profoundly weaker tremorgen than **62**, suggesting that the C-15 hydroxyl group plays a pivotal role on the tremorgenic effects in this class of compounds. Other semisynthetic analogs, compound **63**, **84**, **87** and **88**, cause no tremors in mice [73]. In addition, reductions in the carbonyl of penitremones A and B with NaHB result in losing activity at the same dose. These findings point to the importance of the carbonyl group at C-10 for related tremorgenic activity in the penitremone carbon skeleton [76]. The structure–activity relationship is presented in Figure 20.

In comparison with the structure of lolitrem B, terpendole C, which only lacks the A/B rings, has a shorter acting tremorgen. It has been reported that the tremorgenic activity of terpendole C is much higher than that of terpendole M in a standard mouse bioassay. Possibly, the 14-OH group interferes with the binding of terpendole C to the corresponding tremorgen receptor, thereby reducing the activity [100]. The hydroxyl group at the C-13 position of the diterpenoid nucleus is critical for inducing tremors, which further substantiates that the tremorgenic activities of compound **156** and **158** are the weakest compared to compound **157**, paxilline and terpendole C and K [144].

Moreover, it has been corroborated that the addition of the A/B rings of janthitrem A and B, distinguished from lolitrem B, are not sufficient for inducing duration tremors. The tremorgenic potency of janthitrem B in mice is determined, which elicits a characteristic tremor response accompanied by incoordination and hypersensitivity to sound and touch in mice [87]. Janthitrem A is more active than B, where a higher tremorgenic potency by janthitrem A should be attributed to the 11,12-epoxy group.

Taken together, many of studies associated with the lolitrem and paxilline derivatives have identified that a great deal of structural features of them necessary for tremorgenic activity. The acetal-linked isoprene unit and the stereochemistry at the A/B ring junction are essential regarding lolitrems characterized by the slow onset and long duration of tremors. By contrast, paxilline compounds containing a α-orientated hydroxyl group at the C-13 position and the influential C-10 functionality are relatively essential for eliciting the fast onset and short duration of tremors [145]. Every type of indole diterpene may exert its effects through binding to different target sites, which needs to be explored through further research.

### 4.3. Anti-Insect Activity

**Figure 21:**
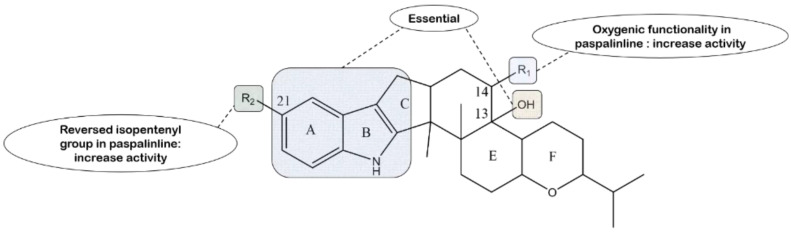
Structure–activity (anti-insect) relationship of paxilline indole diterpenes.

From results in dietary assays against the larvae of *Carpophilus hemipterus* (dried-fruit beetle) and *Helicoverpa zea* (corn earworm), compound **2**, lacking a hydroxyl group at C-13, has essentially no effect compared to paxilline, and the indole-ring containing paxilline is significantly more active than its ring-opened counterpart **28**, implying that the hydroxyl group at the C-13 position and the indole nucleus are essential for eliciting effects [93]. The incorporation of compound **40** into a standard test diet at 100 ppm causes a reduction of 91% in weight gain against *H. zea* relative to the controls. Compound **42** has shown virtually the same result (88%). Interestingly, paspalinine is inactive in an assay at the same level. It is worth further studying whether the oxygenic functionality at C-14 in paspalinine affects the activity [61]. *β*-Aflatrem, an isomer of aflatrem, displays significant activity against the corn earworm *H. zea*, causing a 57% reduction in weight gain over the control at 100 ppm, indicating that the reversed isopentenyl group substituted at the C-21 position of the indole ring can increase the activity in paspalinine skeleton compounds [146]. The corresponding structure–activity relationship is displayed in Figure 21.

**Figure 22:**
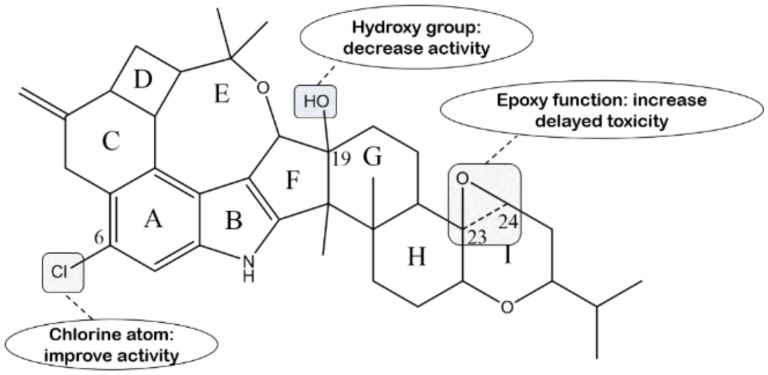
Structure–activity (anti-insect) relationship of penitrem indole diterpenes.

In a study about the insecticidal activity of penitrem compounds against third-instar nymphs of *Oncopeltus fasciatus* and the adults of *Ceratitis capitata*, penitrems A–D and F showed obvious convulsive activity and the inducement of death, whereas penitrem G and paspaline were inactive against both species. Against *O. fasciatus*, the relative toxicity that was displayed generally had a range of penitrem A > F > C > B > D. Against *C. capitate*, the relative toxicity of the penitrems, taking into account the mortality data, had a range of penitrem C > F > A > B > D [147]. It is worth noting that compound **77** exhibits potent activity against *H. zea* with a 95% reduction in weight gain at 100 ppm, which is approximately comparable to that of the pesticide malathion [71]. From these results, we can conclude that the chlorine atom and epoxy function appear to be implicated in acute mortality and delayed toxicity, respectively. On the other hand, penitrem G is the only inactive penitrem, suggesting suppression of the insecticidal activity of the 19-hydroxy group in penitrems [147]. This deduction is further corroborated by compounds **59** and **60**, penitrem A and penijanthine A, which were examined displaying remarkable toxicity against brine shrimp (*Artemia salina*). It is obvious that the Cl-substitution at C-6 tends to strengthen the activity. On the other hand, the hydroxy group at C-19 suppresses the toxicity in penitrems [77]. The structure–activity relationship is shown in Figure 22 characteristically.

Janthitrem A has been shown to have more potent antifeedant activity than B against porina (*Wiseana cervinata*) larvae. By comparing them, it is revealed that greater anti-insect activity should be attributed to the presence of an epoxy group in janthitrem A rather than a double bond. However, structurally related compounds shearinines A–C can reduce the feeding of the dried fruit beetle at 100 µg/g. It appears that shearinine A can reduce the weight gain of corn earworms, and shearinine B can also severely diminish the survival and feeding of fall armyworm larvae. Moreover, shearinine B is apparently more active than C. Although janthitrems and shearinines share the same skeleton, the minor distinction in their terminal H-rings may account for both of them showing anti-insect activity [90,93].

In an assay of comparing the nodulisporic acid A with several known insecticides, it was demonstrated that nodulisporic acid A with an LC_50_ of about 1 ppm is significantly more potent than abamectin and some other common insecticides [148]. Nodulisporic acid A1 was tested with an LD_50_ of 0.3–1 μg/mL against *Lucilia*, similar to that of A. Nodulisporic acid A2 was slightly less active than them [127]. It is likely that the hydration of the side-chain double bond led to a weak impact on the activity. The nodulisporic acids were also examined in an ex vivo flea (*Ctenocephalides felis*) artificial membrane feeding assay. Moreover, their abilities to replace the binding of a [35*S*]-labeled nodulisporic amide derivative were tested in a competition binding assay. According to the results, the nodulisporic acids containing the dienoic acid chain showed better activity than other compounds in their own series, but the D series was the exception. The structural simplicity of nodulisporic acids tended to attenuate the biological activity and bring adverse effects on the binding activity. In general, the activities of Δ23 1″,2″-dihydro nodulisporic acids were significantly lower than corresponding nodulisporic acids of the same series [129].

It was documented that compound **189** had the capacity to completely inhibit feeding by the fungivorous beetle *C. hemipterus* (nitidulidae) at 100 ppm, which also possessed entomotoxicity to the crop pest *H. zea*, identical to that of rotenone. Compounds **190**–**192** were all inactive against *C. hemipterus* at 100 ppm, only showing feeding deterrence when tested [112]. Additionally, compound **196** was found to exhibit evident activity against *H. zea* and *C. hemipterus* in feeding trials, which caused a weight reduction of 68% in *H. zea* and a 38% reduction in feeding rate against *C. hemipterus*. Despite compound **193** and **194** both having a structure close to that of **196**, they did not show any anti-insect effects [113]. Another assay of compound **194** and **197** against *C. hemipterus* and *H. zea* showed little or no activity in either insect dietary tests [114]. Hence, the hydroxyl group at C-20,25 was indispensable for aflavinine compounds to take effect.

With respect to other indole diterpenes, there are also many studies associated with them. Research has shown that asporyzins A and B, as the oxidized structures of JBIR-03, possess lower activity than their precursor JBIR-03 against brine shrimp, which exhibits the highest insecticidal activity, suggesting the importance of the complete indole and additional tetrahydrofuran units [121]. Sulpinines A-C, secopenitrem B and penitrem B are all effective on larvae of the corn earworm *H. zea*. Sulpinine A possesses the strongest activity indistinguishable from the commercial pesticide permethrin, and sulpinine C has the weakest. Apparently, the unusual eight-membered-ring lactam of sulpinine C decreases the anti-insect activity enormously [56]. Radarin A can induce a 52.7% reduction against the corn earworm *H. zea* in weight gain relative to the control. Radarin C exhibits moderate activity, causing 17.1% reduction, and radarins B and D are inactive at the same concentration [136]. In terms of the radarins, the results suggest that the carbonyl group in the diterpenoid moiety is necessary for inducing anti-insect activity, and the hydroxyl group of the indole unit is likely to promote the effect synergistically.

### 4.4. Antimicrobial Activity

From an assay of compound **15**, **50**, *β*-aflatrem and paspalinine for antibacterial activity against *Enterobacter aerogenes*, *Bacillus subtilis*, *Escherichia coli*, *Pseudomonas aeruginosa*, *Staphylococcus aureus* and *Candida albicans*, only **50** displayed antibacterial activity against *S.aureus* with a MIC value of 20.5 μM [47]. Against aqua-(*Edwardsiella tarda* and *Vibrio anguillarum*) and human pathogens (*E. coli* and *S. aureus*), compound **59** and **60**, as well as penitrem A, possessed mild activity, and penijanthine A was inactive. Interestingly, the chlorinated compounds exhibited better activity, suggesting that the chlorine atom was crucial to the antibacterial activity [77]. However, it is puzzling that, in another screening of natural compounds for their anti-*Vibrio* activity, penijanthine A was found to display strongest effect against *V. Anguillarum*, *V. parahemolyticu*, and *V. Alginolyticus*. Penijanthine B showed relatively moderate activity towards three pathogenic *Vibrio* spp., indicating that the presence of an acetoxy group at C-7 in penijanthine B may attenuate the anti-*Vibrio* activity [64]. The related outcome should be verified in further studies.

It was confirmed using standard agar diffusion tests that only asporyzin C had powerful activity against *E. coli*, and none exhibited any antifungal activity for six compounds (asporyzin A-C, JBIR-03, emindole SB and emeniveol). We can deduce that the 4-hydroxy-4-methylpent-2-enyl moiety in asporyzin C may be critical for the antibacterial activity against *E. coli* [121].

Drechmerins A-G, along with terpendoles A, C, I and compound **2**, were evaluated for their antimicrobial activity against *C. albicans*, *S. aureus*, *B. cereus*, *B. subtillis*, *P. aeruginosa* and *K. pneumonia*, respectively. Drechmerin B displayed the strongest antimicrobial activity against *C. albicans*, and drechmerins A, C, G and terpendole I showed weak antimicrobial effects, whereas the remaining compounds were all inactive. The results of molecular docking revealed that drechmerins A–C, F, G and terpendole I were fitted the ligand binding domain of peptide deformylase (PDF), but drechmerin E and terpendole C did not. Drechmerins A and B could interact with catalytic metal ion Zn2202 and Cys-111 as opposed to drechmerins C and F, revealing that a large moiety replaced in indole diterpenes is unfavorable to antimicrobial effects, which is a conclusion that is in line with in vitro antimicrobial experimental results [107]. For *C. albicans*, *S. aureus*, *B. cereus*, *B. subtillis*, *P. aeruginosa* and *K. pneumonia*, drechmerin I displayed an inhibitory effect only against *B. subtillis*. Furthermore, an investigation regarding interactions between it and PDF, which was regarded as a potential target of antimicrobial drugs using molecular docking, revealed that indole diterpenes could be docked into the catalytic site of PDF entirely through hydrogen bonds with Try-88 and Arg-143 [106].

Tolypocladins A-J were evaluated for their activity against ten agricultural pathogenic fungi and eight human pathogenic bacterial strains. Only tolypocladin A showed significant inhibitory activity toward seven pathogenic fungi, and tolypocladin H was active toward all tested bacteria. From the results, the compounds had high selectivity for different microbes, and the prenylated indole nucleus may play an important role for the antimicrobial activity of tolypocladins [109].

### 4.5. Modulation of Maxi-K Channel Activity

A number of studies have shown that indole diterpenes are the most potent nonpeptidyl inhibitors of maxi-K channels. Paxilline, paspalinine, aflatrem, penitrem A and paspalitrem A and C could all inhibit maxi-K channels in electrophysiological experiments. In terms of paxilline, it was inactive when the carbonyl group at C-10 was substituted by an amino group. The double bond of the indole B ring and hydrogen at N-1 were both substituted by a hydroxyl group to produce 1,18-dihydroxy-2-methylpaxilline, which was devoid of activity [53].

The maxi-K channel inhibitory activities of penitrem A, emindole SB and compounds **62**, **63**, **84** and **87** were all estimated by the *C. elegans* model in vivo. Compound **62** was similar to penitrem A, indicating that halogenation had no effect in inhibiting the maxi-K channel. In addition, other compounds scarcely displayed inhibitory activity in this assay [72,73].

In comparison to compound **105**, lolitrems B, E, F and M and lolitriol, the stereochemistry of the A/B ring junction and the isoprene moiety were demonstrated as major structural determinants by lolitrem B for inhibiting BK channels. Adding an acetate a moiety to the free 10-OH group of lolitrem E and F can increase their potencies [149].

As reported from shearinines D-K, with blocking efficacies at 0.1 and 1 μM, the IC_50_ values for shearinine D and E were 150 nM and 170 nM, respectively, which were approximately 10-fold higher than that for the positive control penitrem A (17 nM). By contrast, shearinines F and H-J exhibited only negligible effects. Thus, the data clearly reveal that the replacements of C-22, similar to those of C-13, are essential determinants for BK inhibition by janthitrems [66].

### 4.6. Inhibition of ACAT Activity

The ACAT inhibitory activities of emindole SB, paspaline and terpendoles A–D were measured both in an in vitro enzyme assay and a cell assay. Among the four terpendoles, C was the most efficient in the enzyme assay, and D exhibited the highest specificity, which was based on the cholestery ester formation inhibitory activity and the cytotoxicity in the J774 macrophages assay. Emindole SB exhibited moderate inhibitory activity, and paspaline almost lacked that [98]. Subsequently, terpendoles E–L were isolated, and the inhibitory activity of ACAT was also evaluated by the same method mentioned above. Terpendoles J–L exhibited moderate inhibitory activity, but the effects of others could be neglected [97]. Considering that the data originated from different literature, the results were discussed separately, which suggested that an ether-linked isoprene unit linked to the indoloditerpene core was the primary functional group of terpendoles for the inhibitory effect on ACAT.

**Figure 23:**
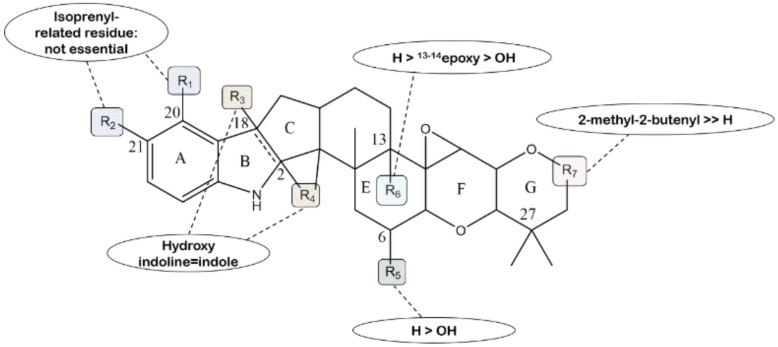
Structure–activity relationship of terpendole indole diterpenes.

The latest research about the human sterol *O*-acyltransferase (SOAT) inhibitory activity of eleven terpendole compounds shows that terpendoles O and P exert inhibition effects on both SOAT1 and SOAT2 isozymes to a similar extent, and terpendole N has no effect against both isozymes. Based on the common terpendole skeleton structure, the structure–activity relationship is illustrated in detail (Figure 23). Firstly, consecutive rings, including an indole or indoline, maintain the cornerstone for SOAT inhibitory activity because the opened ring A leads to the loss of activity in terpendole N. Secondly, the comparison among terpendole D, J, L, O and P; voluhemins A and B; and NK12838 shows that the presence of an isoprenyl-derived moiety at the C-20 and C-21 positions is not important for the activity. Thirdly, the hydroxyl group at the C-18 and C-2 positions in voluhemin A and NK12838 has no impact on the activity. Fourthly, the presence of a proton at the C-6 and C-13 positions can enhance the SOAT inhibitory activity by comparing terpendole D, J and P; voluhemin A; and NK12838. Finally, a free hydroxyl group at the R7 position leads to the loss of SOAT inhibitory activity from the results of terpendole D, J and P and tolypocladin A [103].

Additionally, Ohshiro et al. found that voluhemin A and NK12838 exert inhibition effects on both SOAT1 and SOAT2 isozymes, and voluhemin B shows SOAT2 selective inhibition. By considering the structural differentiation of the three compounds, we can find that a methoxy group at the hemiaminal position in voluhemin B is key to displaying the SOAT2 selective inhibitory effect [104].

### 4.7. Others

Some indole diterpenes with other different activity are continuously being investigated, such as emeniveol, which is able to inhibit pine pollen germination and tea pollen growth, and shearinine L, which can interfere with the normal behavior of *Acromyrmex octospinosus* ants [96,120]. Paxilline, compound **13** and pyrapaxilline have been screened to find the inhibition effects of LPS-induced NO production for further potent anti-inflammatory agents, which shows that isopentenyl at C-21 or an additional dihydropyran ring fused to the indole moiety both can presumably lead to a decrease in activity associated with the modulation of the maxi-K channel [92]. Emindole SB and penerpenes A and B have been demonstrated to have great potential for inhibiting nontransmembrane protein tyrosine phosphatases (PTPs). Further molecular docking experiments have suggested that penerpene A can bind deeply in the active site pocket via forming H-bonds with Asp-181 and Gln-262, and penerpene B does not bind to the active site but instead interacts with Phe-30 at the secondary bonding site of PTP1B for similar activity with penerpene A [57,134,135].

## 5. Conclusions and Future Perspectives

With the view of key enzymes for biosynthesis and structure–activity relationships, this review provides new insight for the further industrial-scale production of indole diterpenes and new inspiration for developing new agents for clinical prevention and treatment against related diseases. Through the study of indole diterpenoid biosynthetic enzymes, chemical methods have been used to introduce clusters into the compounds to enhance affinity and bioavailability to drug targets, thus improving the efficacy of drugs. However, due to the complexity and diversity of natural products, it is still difficult to modify functional groups by chemical methods.

The heterosis of enzymes has attracted more and more attention, and this is of great significance for the development of tool enzymes for the synthesis of pharmaceutical active compounds and for the introduction of isopentenyl groups into the study of synthetic biology, thus providing a rich and innovative compound for the development of new drugs. However, the research on biosynthesis enzymes is still immature; for example, the biosynthesis pathways of compounds without regularity are still difficult. The mechanism of some discovered enzymes is still unclear, and the effect of the enzymes’ spatial structures on their catalysis is yet to be studied. Therefore, more attention should be paid to the related research, to the large-scale production of target indole diterpenes by gene prediction and biomolecular cloning techniques and to mining engineering bacteria with a superior ability to produce the target compounds in large quantities, thus providing a more general approach for the synthesis of the natural products of the family. Thus, the economic value and social utility of indole diterpenes can be brought into full play.

The sources recognize that indole diterpenoid compounds originate from natural fungi or endophytic fungi in plants. Nevertheless, it is noted that there are only few reports referring to indole diterpenes derived from plants directly, except for *I. asarifolia* and *I. muelleri* [106]. By considering the *Ipomoea* species containing ergot alkaloids, it has been proposed that indole diterpenes can also exist in various plants with similar neurotoxic alkaloids [150]. On the other hand, the chemical composition of host plants corresponding to indole-diterpene-rich endophytic fungi should be investigated thoroughly. How to explain beneficial relationships between endophytic fungi and medicinal plants through indole diterpenes is a valuable subject. In light of new compounds reported in recent years, an ever-growing array of indole diterpenoid natural products has been discovered. The acquisition of some new scaffold structure compounds deformed from indole diterpenes such as penerpenes, tolypocladins, drechmerins, etc., has demonstrated the abundant species diversity of this type of compound. More and more indole diterpenes containing unique scaffolds or structure units are deserving further exploration.

It is widely known that indole diterpenes such as lolitrem B and penitrem A are prime causes for the “ryegrass staggers” disorder. Although most indole diterpenes have been regarded as a tremorgenic mycotoxin over the years, their great potential in medical applications on account of their potent and wide-ranging bioactivity should not be neglected. The prevention and treatment of malignant tumors is still recognized as a worldwide difficulty, and it is also one of the most serious threats to health for people [151]. The discovery of natural antitumor products, such as paclitaxel and vincristine, is always one of the most important strategies for overcoming cancer. Paclitaxel and vincristine, both as terpenoid indole alkaloids with low content in plants, have been approved for the treatment of cancers by the FDA in the last century [152]. They are both widely used in clinics, representing highly efficient tubulin inhibitors [153]. Likewise, indole diterpenes are rarely separated from plants, perhaps due to low content. Considering the similar structural combination between terpenoids and indole units, indole diterpene compounds are speculated to possess excellent antitumor activity through inhibiting the depolymerization of microtubules and making cell division stop in mitosis, which needs further experiment to verify. At present, research about the antitumor activity of indole diterpenes still remains mainly in the in vitro stage; therefore, the primary mechanism is also unclear. Moreover, remarkable anti-insect activity, such as that demonstrated by shearines and nodulisporic acids, makes it possible for them to become candidates for pesticides used in agriculture. Indole diterpenes with striking antimicrobial effects, including penijanthines, asporyzins, drechmerins and tolypocladinscan, can be focused on in analyses of resistance in further studies in order to obtain ideal antimicrobial agents. The modulation of the maxi-K channel activity of penitrems, lolitrems and shearinines may be associated with their tremorgenic effects and cytotoxicity, which require more detailed research to describe. The inhibition of ACAT activity is conducive to developing new drugs for indole diterpenes to prevent atherosclerosis, hypercholesterolemia and fatty liver diseases [154,155,156,157]. Furthermore, in the chemical class of drugs, the vast majority of active ingredients are natural lead compounds, which need to be further modified in order to achieve medicinal purposes. Thus, this is indispensable work to analyze and summarize comprehensively. The structure–activity relationship, which has established the cornerstone of designing and selecting the most appropriate indole diterpenes with high efficacy and low toxicity, can be used for synthesizing in clinical trials. This review accounts for the lack of integrated data associated with the structure–activity relationship for indole diterpenes. Taken together, indole diterpenes are a class of valuable compounds that need to be deeply studied, which should bring hope to people for the therapy of various diseases in the future.

## Figures and Tables

**Table 1:** Antitumor activity of indole diterpenes (IC_50_, μM). Reproduced with permission [67]. Copyright 2016, American Chemical Society.

Compound	A549	HL-60
**78**	11.5	9.6
**79**	6.3	5.0
**82**	9.2	-
**54**	8.4	7.0
**67**	8.0	4.7
**57**	8.2	3.3
**6**	4.6	2.6

“-” means not reported.

**Table 2:** The cytotoxicity (IC_50_ in μM) of several compounds towards the L5178Y, A2780, J82 and HEK-293 cell lines. Reproduced with permission [48]. Copyright 2019, American Chemical Society.

Compound	L5178Y	A2780	J82	HEK-293
**44**	5.3	12.2	42.1	21.7
**19**	5.3	-	ND	27.9
**12**	-	12.2	55.3	ND
**134**	-	32.2	96.7	ND
**135**	8.1	7.8	31.7	37.4
**136**	7.6	11.9	29.4	28.3
**139**	-	19.4	73.0	ND
**137**	-	51.5	-	ND
**203**	18.3	8.2	ND	44.6
**13**	12.9	-	ND	ND
**2**	-	17.1	ND	ND
**35**	-	5.3	ND	43.0
**5**	6.2	-	ND	39.8
**6**	-	28.5	ND	ND
**138**	10.9	12.8	ND	ND
**45**	-	19.8	ND	ND

“-” means inactive; “ND” means not detected.

**Table 3:** Cytotoxity of anthcolorin compounds (IC_50_, μM). Reproduced with permission [132]. Copyright 2013, Elsevier.

Compound	P388 Cell Line
**260**	17.4
**261**	8.5
**262**	2.2
**263**	5.5
**264**	22.1
**265**	26.7

## Data Availability

Not applicable.

## Sample Availability

Samples of the compounds are not available from the authors.
